# Effects and Mechanism of Different Phospholipid Diets on Ovary Development in Female Broodstock Pacific White Shrimp, *Litopenaeus vannamei*

**DOI:** 10.3389/fnut.2022.830934

**Published:** 2022-02-18

**Authors:** Xiaolong Liang, Xiaolong Luo, Hongxing Lin, Fenglu Han, Jian G. Qin, Liqiao Chen, Chang Xu, Erchao Li

**Affiliations:** ^1^Key Laboratory of Tropical Hydrobiology and Biotechnology of Hainan Province, Hainan Aquaculture Breeding Engineering Research Center, College of Marine Sciences, Hainan University, Haikou, China; ^2^School of Biological Sciences, Flinders University, Adelaide, SA, Australia; ^3^School of Life Sciences, East China Normal University, Shanghai, China

**Keywords:** *Litopenaeus vannamei*, broodstock, phospholipids, gonadotrophin, ovarian maturation

## Abstract

Research on nutrition and feed development for the broodstock of the Pacific white shrimp, *Litopenaeus vannamei*, is rare, and a poor broodstock quality is a critical factor restricting the seed supply in shrimp farming. As an essential nutrient for the gonadal development of *L. vannamei*, one control diet (no phospholipid) and three typical phospholipids (soybean lecithin, egg yolk lecithin, and krill oil) were evaluated in a semipurified diet of 4% phospholipid for a 28-day trial (initial weight 34.7 ± 4.2 g). Dietary phospholipid supplementation significantly promoted the ovarian maturation of female *L. vannamei*. Compared with soybean lecithin and egg yolk lecithin, krill oil showed the best positive results. Shrimp fed with a diet krill oil has obtained a significantly higher gonadosomatic index, yolk particle deposition, lipid accumulation, and estrogen secretion than from other sources. Ovary lipidomic analysis showed that the krill oil enriched the lipid composition of the ovary. The “glycerophospholipid metabolism” and “sphingolipid metabolism” pathways were significantly varied *via* topological pathway analysis. Genes and hub genes, with significantly different expression levels, were significantly enriched in the “fatty acid metabolism pathway,” “glycerophospholipid metabolism,” and “arachidonic acid metabolism” pathways by transcriptomic analysis. Correlation analysis of the transcriptome and lipidomics showed that the differential gene “hormone-sensitive lipase-like” (*HSL*) was positively correlated with various lipids [triglycerides (TG), phosphatidic acid (PA), phosphatidylserine (P), phosphatidylethanolamine (PE), glucosylceramide (GlcCer), phosphatidylglycerol (PG), and phosphatidylinositol (PI)] but was negatively correlated with diacylglycerol (DG), lysophosphatidylethanolamine (LPE), and sphingomyelin (SM). In conclusion, the dietary phospholipids, especially krill oil as a phospholipid source, can promote the development of *L. vannamei* ovaries by increasing the accumulation of nutrients such as triglycerides and sterols, and the secretion of estrogen or related hormones, such as estradiol and methylfarneside, by affecting the metabolism of glycerol phospholipids and some key fatty acids.

## Graphical Abstract

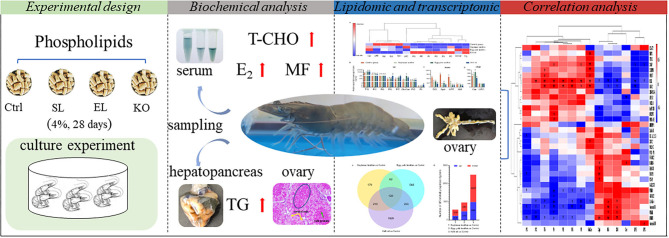

## Introduction

The Pacific white shrimp *Litopenaeus vannamei* (Boone, 1931) is a species native to the Pacific coast in Central and South America (1). The production of *L. vannamei* (4.9 million tons) in the world accounted for 82.8% of the world's total crustacean production in the aquaculture sector in 2018 (2), which is one of the most economically important marine crustaceans farmed in the world. Given the degradation of germplasm resources (3), the broodstock of *L. vannamei* is currently based on breeding under biosafety conditions over the past few years (4, 5). Due to the lack of suitable dedicated diet nutrition, the reproductive performance of artificial culture was lower than that of broodstock captured from the natural environment (6).

Traditionally, the rearing of artificial culture broodstock still relies on a variety of fresh and frozen foods, such as marine polychaetes and squid (7). Due to the risk of disease transmission and unstable nutritional quality, the use of fresh food is considered to be unsuitable for the sustainable development of the shrimp breeding industry (5, 8, 9). The research and development of a fully nutritious compound feed can effectively promote a gonad maturation, improve the quantity and quality of larvae, and benefit the development of the industry because of its precise nutritional composition, long shelf life, stable supply, and low risk of contamination (10). Thus, it is necessary to conduct a nutrition study and to develop a specific diet for *L. vannamei* broodstock.

Lipids in natural biological foods contain various functional components, such as phospholipids, which can induce a secondary vitellogenesis and stimulate ovarian maturation in a female shrimp (7, 11, 12). Phospholipids are composite lipids containing phosphorus, such as phosphatidylserine (PS), phosphatidylinositol (PI), phosphatidylethanolamine (PE), and phosphatidylcholine (PC) (13). Phospholipids are the predominant ingredients in shrimp ovaries, which are mainly PC (75–80%) and PE (20–25%) (14, 15). Phospholipids can be biosynthesized in most crustaceans, however, the synthesis efficiency is relatively insufficient, especially during gonad maturation of broodstock shrimp (16). In a study of *Macrobrachium rosenbergii*, dietary phospholipids had a positive effect on ovarian development and reproductive performance, and the results showed that 2% of dietary soybean lecithin could meet the needs of a female sexual maturity (17). In the study of *Eriocheir sinensis*, the highest value of ovarian gonad index was found in crabs fed with a diet of 2.4% soybean lecithin (18). Similarly, 2% soybean lecithin could upregulate the expression of the vitellogenin (Vg) gene in the hepatopancreas of *Cherax quadricarinatus and L. vannamei* (19, 20). However, previous studies on phospholipids in aquatic animals mainly focused on nutritional requirements, and soybean lecithin was used as the sole phospholipid source in the diet. Although the main nutrients of different phospholipid sources are different (total phospholipids, 10–33% PC and 9–23% PE in soybean lecithin, 65–76% PC and 9–24% PE in egg yolk lecithin, and 87.93–95.16% PC and 4.84–12.07% PE in krill oil) (21, 22), there is no information, so far, about the effects of different phospholipid sources on ovary development and on reproductive performance of *L. vannamei*. At present, studies on lipid metabolism and ovarian development of crustaceans fed with diets with different phospholipid sources (e.g., soybean lecithin, egg yolk lecithin, and krill oil) during the pre-reproductive phase have only been conducted in *E. sinensis* (24). In addition, the physiological and molecular mechanisms of phospholipids promoting a gonadal development in crustaceans have not been evaluated.

This study aims to identify the suitable source of phospholipids from three ingredients (soybean lecithin, yolk lecithin, and krill oil) for Pacific white shrimp broodstock. Lipidomics and transcriptomics were used to investigate the effects of dietary phospholipids on endocrine hormones, ovarian development, and lipid metabolism in developing gonads in shrimp. In addition, appropriate phospholipid sources were screened for the development of gonads in *L. vannamei*, and the effects and mechanisms of phospholipids on gonads were clarified.

## Materials and Methods

### Experimental Diets

According to the broodstock nutrient requirements of *L. vannamei* (20, 25), four diets with 52.4% crude protein and 14.2% crude lipid were formulated containing 4% soybean lecithin (PC + PE >70%), egg yolk lecithin (PC + PE >80%), or krill oil (PC + PE >40%). A diet without phospholipid supplementation was set as the control in the experiment. Dietary crude protein and crude lipid contents were detected by the methods of Dumas combustion and Soxhlet extraction, respectively (Table 1). Fish meal, gelatin, and casein were used as protein sources. Cholesterol, palm oil, fish oil, and phospholipids served as lipid sources in the formula. The granular raw materials were ground by a grinder and then crushed into powder through an 80-mesh sieve. All raw materials were added and were fully mixed before the liquid ingredients (oils and double distilled water) were added to the dry ingredients. This dough was turned to a double helix grinder (model: CD4-1TS) to extrude into particles with a diameter of 2.5 mm and dried at room temperature. All diets were stored in sealed plastic bags at −20°C until use. The fatty acid composition of the four diets is shown in Supplementary Table S1.

**Table 1:** Ingredient formulation (g/kg dry basis) and proximate composition (%) of the four experimental diets fed to female *L. vannamei*.

**Ingredients**	**Experimental diets**			
	**Control**	**Soybean lecithin**	**Egg yolk lecithin**	**Krill oil**
Fish meal	200	200	200	200
Casein	320	320	320	320
Gelatin	80	80	80	80
Corn starch	150	150	150	150
Fish oil	10	10	10	10
Soybean lecithin	0	40	0	0
Egg yolk lecithin	0	0	40	0
Krill oil	0	0	0	40
Cholesterol	5	5	5	5
Palm oil	80	40	40	40
Butylated hydroxytoluene	1	1	1	1
Anhydrous calcium carbonate	4	4	4	4
Calcium lactate pentahydrate	4	4	4	4
Choline chloride	5	5	5	5
Inositol	0.25	0.25	0.25	0.25
Betaine	20	20	20	20
Vitamin premix^a^	10	10	10	10
Mineral premix^b^	20	20	20	20
Carboxymethyl cellulose	20	20	20	20
Cellulose	70.75	70.75	70.75	70.75
Total	1,000	1,000	1,000	1,000
**Analyzed proximate composition (%)**				
Moisture	7.51	7.43	7.78	7.42
Crude protein	52.33	52.15	52.50	52.47
Crude lipid	14.28	14.40	14.05	14.22
Ash	8.31	8.30	8.28	8.31

a *Vitamin premix (per 1,000 g premix): vitamin A acetate (500,000 IU/g), 0.480; L-ascorbyl-2-polyphosphate 35% Active C, 35.710; folic acid, 0.180; 2% biotin, 2.5; riboflavin, 3; DL- Ca-pantothenate, 5; 99% pyridoxine HCl, 1; 1% vitamin B12, 0.200; thiamin HCl, 0.500; menadione, 2; DL-alpha-tocopheryl acetate (250 IU/g), 8; nicotinic acid, 5; vitamin D3 (500,000 IU/g), 0.800; defatted rice bran, 935.630. All ingredients are filled with α-cellulose to 1,000 g*.

b *Mineral premix (per 1,000 g premix): zinc sulfate monohydrate, 20.585; calcium iodate, 0.117; cupric sulfate pentahydrate, 0.625; manganous sulfate monohydrate, 1.625; magnesium sulfatemonohydrate, 39.860; cobalt chloride, 0.010; ferrous sulfate monohydrate, 11.179; sodium selenite, 0.025; calcium hydrogen phosphate dihydrate, 166.442; wheat bran flour, 759.532. All ingredients are filled with α-cellulose to 1,000 g*.

### Feeding Management

The full-sib family of female *L. vannamei* broodstock was obtained from a local private company in Hainan, China. Prior to the feeding trial, shrimp were reared in black circular polypropylene barrels (diameter × height = 5.8 × 1.1 m) and fed with the control diet for 7 days to acclimate to the experimental conditions. After acclimatization, 160 female shrimps (initial weight ranging 34.7 ± 4.2 g, hepatosomatic index ranging 6.23 ± 0.25%, gonadosomatic index ranging 0.51 ± 0.12%) were randomly allocated to 16 barrels (diameter × height = 1 m × 0.9 m), with 10 shrimps per barrel and four replicates for each diet group. According to the feeding conditions of the pre-experiment and reference (26), shrimps were fed seven times daily (7:30, 10:00, 13:00, 15:00, 18:00, 21:00, and 23:30) at 5.5% of their biomass during the 28 days of cultivation. The uneaten food and excrement were removed with a siphon tube twice daily with water renewal (50%) until the end of the trial. The water quality parameters were controlled at 28–29°C, pH 7.8–8.4, salinity 30–32, dissolved oxygen 5–6 mg/L, ammonia nitrogen 0.10–0.30 mg/L, nitrite 0.03–0.10 mg/L, and a photoperiod of 12 h light and 12 h dark throughout the feeding trial.

### Sampling and Calculation

The ovary and hepatopancreas of 15 shrimps were collected before the experiment, and the gonadosomatic index and hepatosomatic index were measured to obtain the initial values. Daily flashlight was used to observe the size and color of the gonads on the backs of the shrimps to evaluate the gonad development (27). The percentage of ovarian development in III-V phase was calculated on the 7th, 14th, 21st, and 28th day. After the 28-day experiment, shrimps were fasted for 24 h and anesthetized in an ice bath for 10 min before sampling. The shrimps in each barrel were weighed and counted. Sampling and analysis were done from ovary development to maturation (IV–V) (26). The hemolymph of shrimp was sampled from the cardio coelom with a 1 ml disposable sterile syringe and stored at 4°C overnight. Hepatopancreas, eyestalk, and ovaries were immediately separated and frozen in liquid nitrogen and then stored at −80°C for later analysis. Before that, the middle lobe of the ovary was fixed in 4% paraformaldehyde for histological observation by hematoxylin-eosin (H&E) staining in paraffin tissue sections. The gonadosomatic index (GSI) and hepatosomatic index (I) were calculated as follows:

Gonadosomatic index (GSI, %) = 100 × (wet ovary weight/wet body weight).

Hepatosomatic index (HSI, %) = 100 × (wet hepatopancreas weight/wet body weight).

### Lipid Metabolism and Hormone Analysis

Eight hemolymph samples per treatment, from two shrimps per barrel, were centrifuged at 1,500 g at 4°C for 10 min. Supernatants were applied to detect the contents of 17-β-estradiol (E_2_), methyl farnesoate (MF), total cholesterol (T-CHO), low-density lipoprotein (LDL-C), and very-low-density lipoprotein (VLDL). The T-CHO and LDL-C determinations were conducted using diagnostic reagent kits (Nanjing Jiancheng Bioengineering Institute, China). The E_2_, MF, and VLDL measurements were performed with enzyme immunoassay kits (Nanjing Jiancheng Bioengineering Institute, China). The specific operation steps were carried out according to the instructions of the manufacturer.

Eight eyestalks and hepatopancreas per treatment, from two shrimps per barrel, were homogenized in a prechilled 0.86% saline solution (1:10, w/v) at a frequency of 60 Hz at 4°C for 30 s (Tissuelyser-24, Jingxin Technology, Shanghai, China) and centrifuged at 1,500 g for 15 min at 4°C (3–18 KS, Sigma, Germany). After centrifugation, the supernatant of eyestalk homogenate was collected to measure the gonad-inhibiting hormone (GIH) and molt-inhibiting hormone (MIH) contents using diagnostic reagent kits (Shanghai Jianglai Biotechnology Co., Ltd., China). In addition, the hepatopancreas homogenate supernatant was collected to measure the triglyceride (TG) content using diagnostic reagent kits (Nanjing Jiancheng Bioengineering Institute, China). The specific operation steps were carried out according to the instructions of the manufacturer.

### Histology of the Ovary

Ovarian tissue samples were fixed in 4% paraformaldehyde solution, which was further dehydrated, cleaned, and balanced with ethanol, toluene, and xylene. Then, the samples were embedded in paraffin and were cut with a rotary slicer (rm2125, Leica, Germany) with a thickness of 5 μm. Ovarian sections were stained with hematoxylin-eosin (H&E) and observed under a microscope (Olympus, model BX51, Tokyo, Japan). Image-Pro Plus 6.0 software was used to calibrate and measure ovarian cells.

### Ovary Lipidomic

Four replicated ovary samples from each experimental group were used for an untargeted LC–MS-based lipidomics detection. A 200 μl sample was extracted with 960 μl extract solution (MTBE: methanol = 5: 1) containing the internal standard. The supernatant was prepared by an ultrasonic treatment and centrifugation. The quality control (QC) sample was prepared by mixing an equal amount of aliquot (10 μl) of the supernatants from all samples, and then 75 μl of supernatant was transferred into LC–MS microvials for further analysis. The LC–MS/MS analyses were performed using an Ultra High-Performance Liquid Tandem Chromatography (UHPLC) system (Ultra High-Performance Liquid Tandem Chromatography Quadrupole Time of Flight Mass Spectrometry, UHPLC-QTOFMS, 1290, Agilent Technologies) with a Phenomen Kinetex C18 column (2.1^*^100 mm, 1.7 μm) coupled to TripleTOF 6,600 mass spectrometry (AB Sciex). The mobile phase A consisted of 40% water, 60% acetonitrile, and 10 mmol/L ammonium formate. The mobile phase B consisted of 10% acetonitrile and 90% isopropanol, which was added to 50 ml 10 mmol/L ammonium formate for every 1,000 ml mixed solvent. The analysis was carried out with an elution gradient as follows: 0–12 min, 40–100% B; 12–13.5 min, 100% B; 13.5–13.7 min, 100–40% B; and 13.7~18 min, 40% B. The column temperature was 45°C. The autosampler temperature was 4°C, and the injection volume was 0.5 μl (positive) or 6 μl (negative).

The raw data files were converted to files in mzXML format using the “msconvert” program from ProteoWizard. Peak detection was first applied to the MS1 data. The CentWave algorithm in X Color Management System (XCMS) was used for peak detection with the MS/MS spectrum. Lipid identification was achieved through a spectral match using the LipidBlast library. Principal component analysis (PCA) and partial least squares discriminant analysis (PLS-DA) were used to find and to exclude the outliers in the samples of each group to ensure the good repeatability and reliability of data within the treatment. The significant difference among groups was defined *via* orthogonal PLS-DA (OPLS-DA) with model variable importance in projection (VIP) > 1, fold change (FC) > 1, and *p* < 0.05. The quantitative value of differential lipid metabolites was calculated by Euclidean distance matrix and was clustered by using the complete linkage method displayed by the thermal map. The clustering heatmaps were made by online software (https://software.broadinstitute.org/morpheus/).

### Ovary Transcriptome

Four ovary sample replicates of shrimps from each experimental group were used for transcriptome analysis. A total amount of 1 μg RNA per sample was used as input material for the RNA sample preparations. Briefly, mRNA was purified from total RNA using poly-T oligo-attached magnetic beads. The RNA integrity was assessed using the RNA Nano 6,000 Assay Kit of the Bioanalyzer 2,100 system (Agilent Technologies, CA, USA). The library preparations were sequenced on an Illumina NovaSeq platform, and 150 bp paired-end reads were generated. The clean data (clean reads) were obtained by removing the reads containing adapters, reads containing poly-N, and low-quality reads from raw data. At the same time, the Q20, Q30, and GC contents of the clean data were calculated.

The index of the reference genome was built, and the paired-end clean reads were mapped to the transcriptome sequence of *L. vannamei* (NCBI TSA accession number: QCYY00000000) (28). The FeatureCounts v1.5.0-p3 was used to count the read numbers mapped to each gene, and fragments per kilobase million (FPKM) of each gene were calculated based on the length of the gene and on the read count mapped to this gene. Before the differential gene expression analysis, the read counts were adjusted by the edgeR program package for each of the sequenced library using a one scaling normalized factor. Then, the differential expression analysis of the two conditions was performed using the edgeR R package (3.22.5). The differentially expressed genes were evaluated by applying the DEseq2 software with *p* < 0.05 and FC > 1. The Cluster Profiler R package was used to test the statistical enrichment of the differentially expressed genes in Kyoto Encyclopedia of Genes and Genomes (KEGG) pathways. Meanwhile, the hub genes in the related pathway were screened in the modules with the strongest correlation with ovarian maturation by a weighted gene co-expression network analysis (WGCNA).

### Integrative Analysis of Lipidomic and Transcriptomics

To explore the metabolic pathways that promote ovarian maturation of crayfish after the feeding diets with different phospholipid sources, all differential metabolites and differentially expressed genes were mapped to KEGG pathways, ensuring the shared KEGG pathways for integrative pathway analysis. The pathway analysis of differential metabolites was performed by MetaboAnalyst 5.0 (https://www.metaboanalyst.ca/) to identify the key metabolic pathways influenced by three different dietary phospholipids with a pathway impact value (PIV) of >0.05 (29). Spearman correlation analysis was performed using an SPSS software (ver. 26.0, SPSS Inc., USA) to show the potential connection among the differential metabolites and differentially expressed genes. This process did not set the correlation coefficients or the *p*-value thresholds. Instead, the heatmap showed the correlation and significant difference. Differences were regarded as statistically significant at *p* < 0.05 and highly significant at *p* < 0.01.

### Statistical Analysis

All data are expressed as the mean ± standard error (SE). Single-factor analysis of variance and Duncan's multiple comparison test were used to determine the significant differences between all the experimental treatments. A *P* < 0.05 indicated that the difference was statistically significant. All statistical analyses were performed using SPSS Statistics 26 (IBM, Armonk, NY, USA).

## Results

### Ovarian Development and Histology

**Figure 1:**
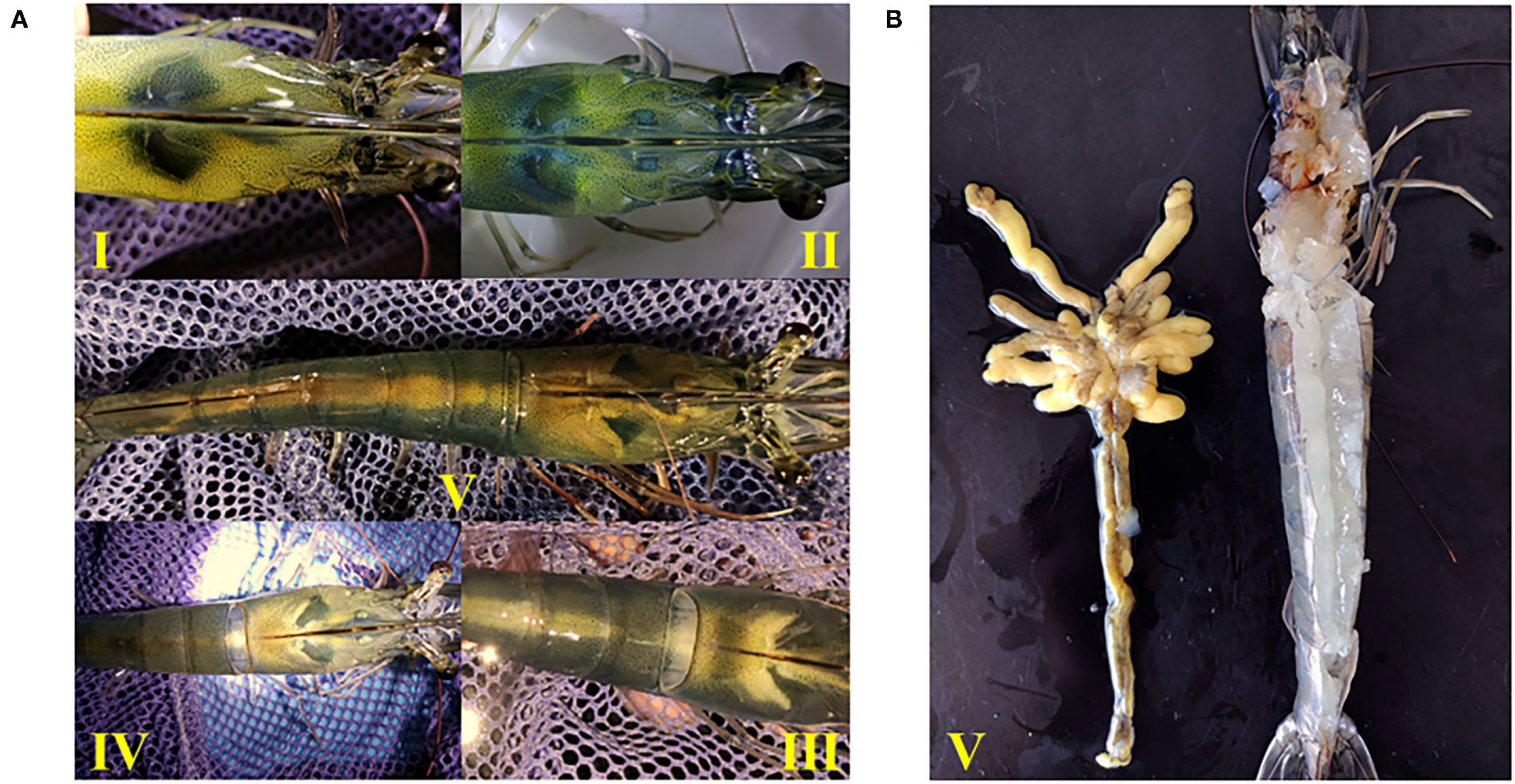
Ovary morphology in different developmental phases of *Litopenaeus vannamei*. **(A)** Phase I-V of ovarian development. I, the ovary is transparent and linear; II, the ovary is translucent or white. III, the ovary becomes enlarged and separates between the body segment and the carapace. IV, the ovary is gradually enlarged and joins between the body segment and the carapace. V, the ovary filled the whole-body segment, the carapace and the lobule extended to the ventral side. **(B)** Anatomical morphology of ovary in phase V.

**Figure 2:**
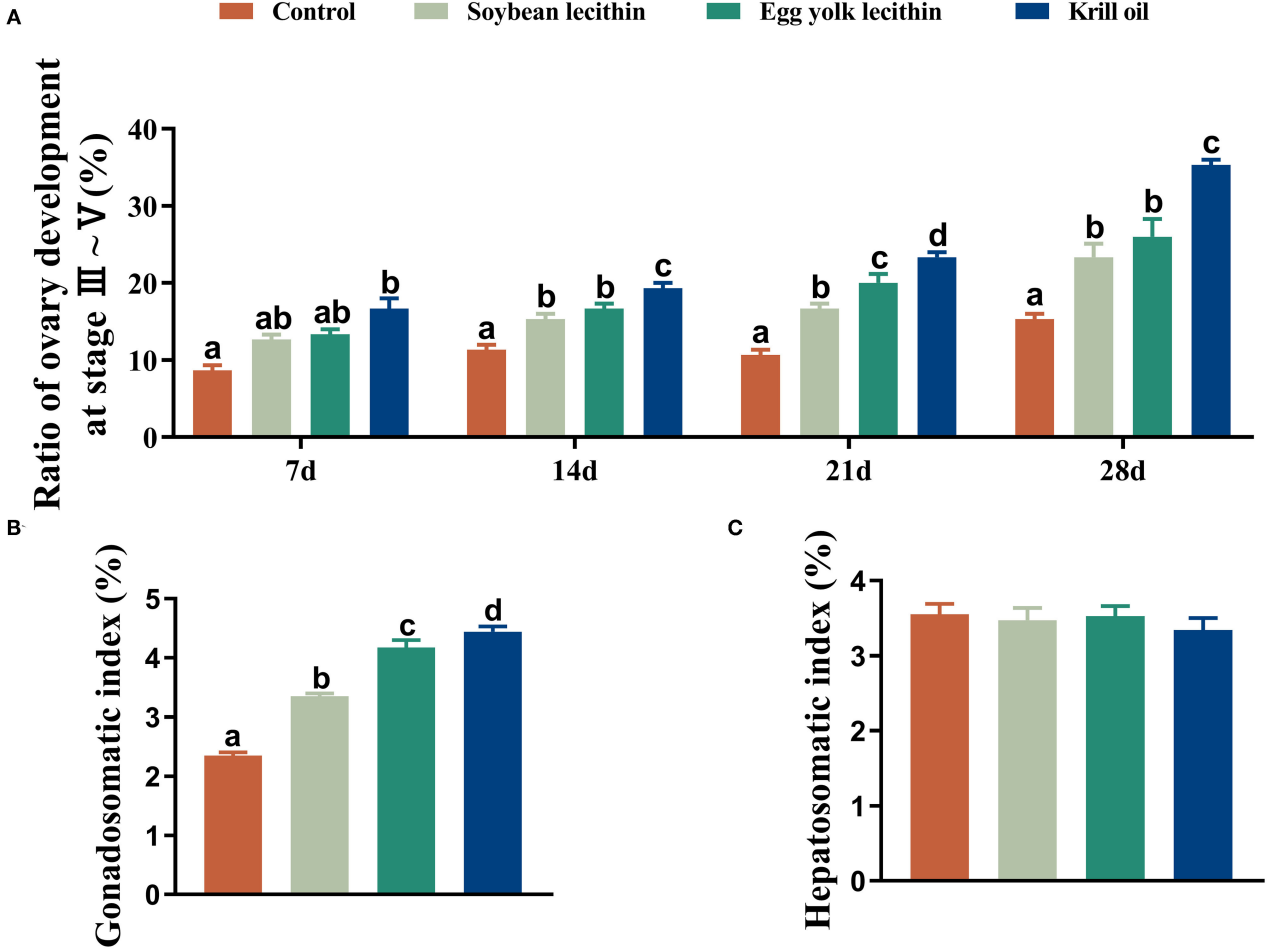
Phenotypic parameters of gonadal development of female *L. vannamei* fed with different experimental diets. **(A)** The proportion of ovarian development from phase 3 to phase 5 at different times. **(B)** Gonadosomatic index. **(C)** Hepatosomatic index. The values are the mean ± standard errors (*n* = 4). Bars with different superscripts are significantly different (*p* < 0.05, single factor analysis of variance and Duncan's tests).

The developmental morphology (I–V phase) of the ovary in shrimp is shown in Figure 1A and the anatomy of the ovary at phase V is shown in Figure 1B. According to the number of ovaries at phase III–V, phospholipid supplementation in the diet has significantly improved the ovary development in broodstock shrimp (*p* < 0.05, Figure 2A). Compared to the control, there was no significant difference in the ovary development status of a shrimp fed with diets with soybean lecithin and egg yolk lecithin within 7 days of the experiment. In contrast, shrimps that were fed with a diet of krill oil showed a better ovarian development status (7 days) (*p* < 0.05, Figure 2A). Within 14 days of the experiment, soybean lecithin and egg yolk lecithin diets played a better role in ovarian development than the control. In comparison, the positive function of krill oil in promoting ovarian development was significantly higher than that of the other experimental treatments (14–28 days) (*p* < 0.05, Figure 2A). At the end of the trial, the gonadosomatic index of broodstock shrimp was significantly enhanced from the initial average value of 0.51 to 3.53–4.44% in all experimental treatments. The highest gonadosomatic index was presented in a female shrimp fed with a diet of krill oil (*p* < 0.05, Figure 2B). The hepatosomatic index of shrimp in all experimental treatments decreased from the initial average value of 6.32 to 3.27–3.64%, and no significant difference was found among all treatments (*p* > 0.05, Figure 2C).

**Figure 3:**
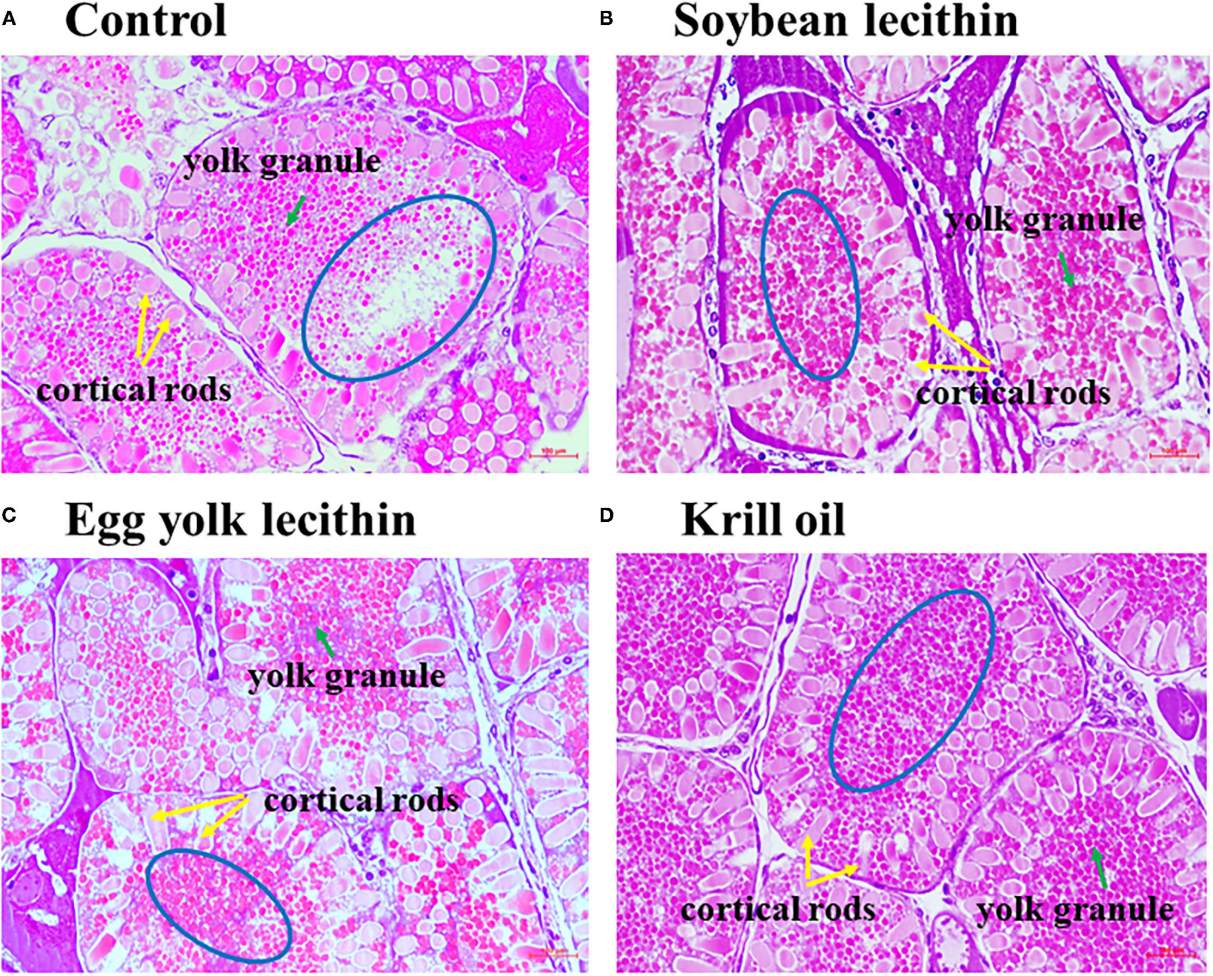
Photomicrographs of ovaries from a shrimp exposed to different treatment diets with hematoxylin and eosin staining to show the changes in yolk granules. Ovarian histology of *C. quadricarinatus* fed with Control diet **(A)**, 4% soybean lecithin diet **(B)**, 4% egg yolk lecithin diet **(C)** or 4% krill oil experimental diet **(D)**. The magnification was 400×, and the scale represents 100 μm. The blue circle emphasizes the deposition density of yolk granules, the green arrow indicates yolk granules, and the yellow arrow indicates cortical rods.

The histology of shrimp ovaries in all experimental treatments showed the characteristics of mature phase (V) that contains mature oocytes fulling cortical rods (Figures 3A–D). In addition, female shrimp fed with diets supplemented with phospholipid showed more yolk granule deposition in the oocytes than the control shrimp. Meanwhile, krill oil and egg yolk lecithin diets can promote yolk granule deposition more effectively than the soybean lecithin diet.

### Lipid Metabolism

**Figure 4:**
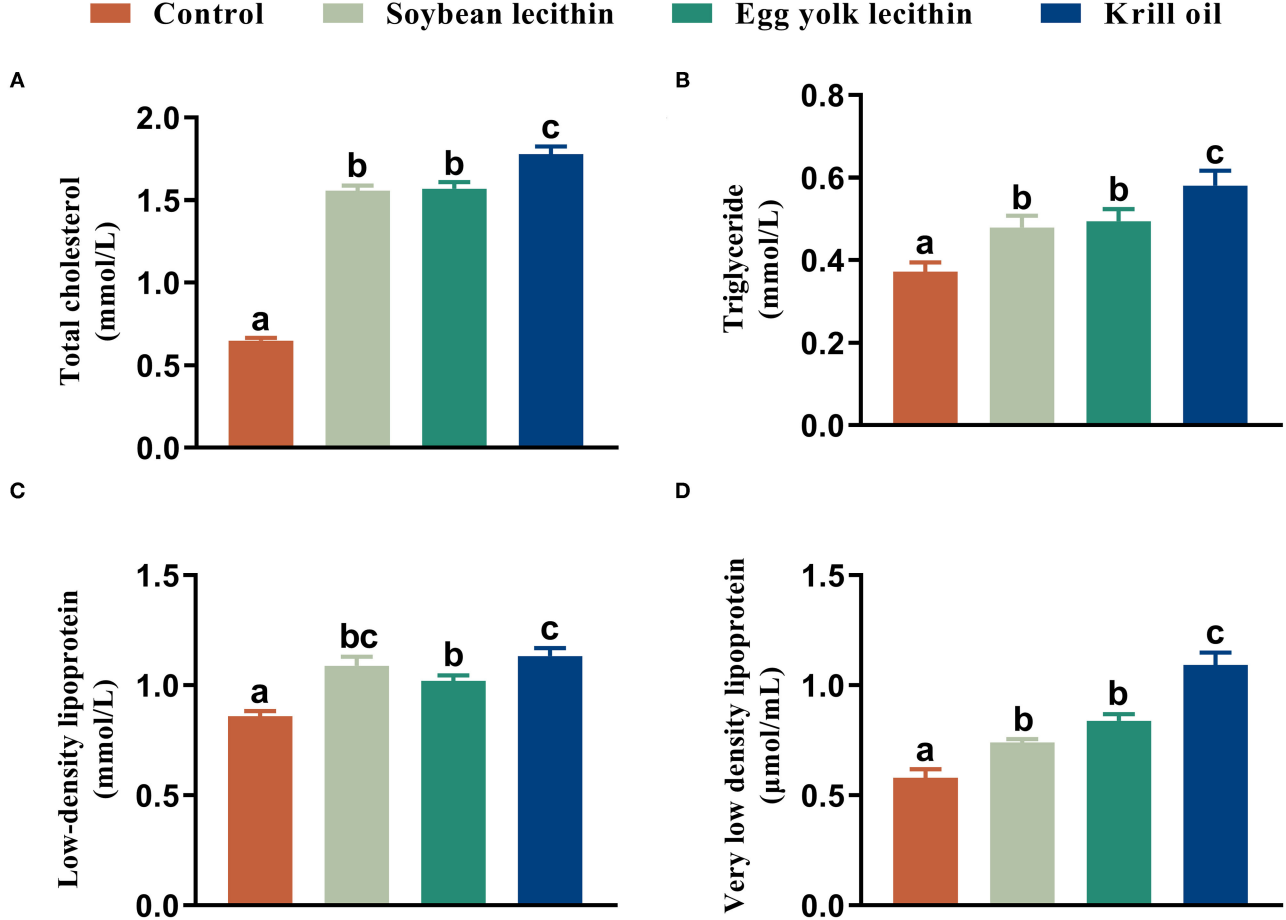
Biochemical composition of female *L. vannamei* fed with different experimental diets. **(A)** Content of total cholesterol in serum. **(B)** Content of triglyceride in hepatopancreas. **(C)** Content of low-density lipoprotein in serum. **(D)** Content of very low-density lipoprotein content in serum. The values are the mean ± standard errors (*n* = 4). Bars with different superscripts are significantly different (*p* < 0.05, single factor analysis of variance and Duncan's tests).

Female shrimp fed with phospholipid diets had a higher total cholesterol content in serum than control shrimp. Among these three experimental treatments, the total cholesterol content in shrimp fed with a diet of krill oil was significantly higher than those in the soybean lecithin and egg yolk lecithin experimental groups (*p* < 0.05, Figure 4A). Similarly, the same results were observed in hepatopancreas triglycerides and in serum with very low-density lipoprotein contents in shrimp (*p* < 0.05, Figures 4B,D). Furthermore, the female shrimp fed with phospholipid supplement diets had a higher serum low-density lipoprotein content than the control shrimp (*p* < 0.05, Figure 4C).

### Hormone Secretion

**Figure 5:**
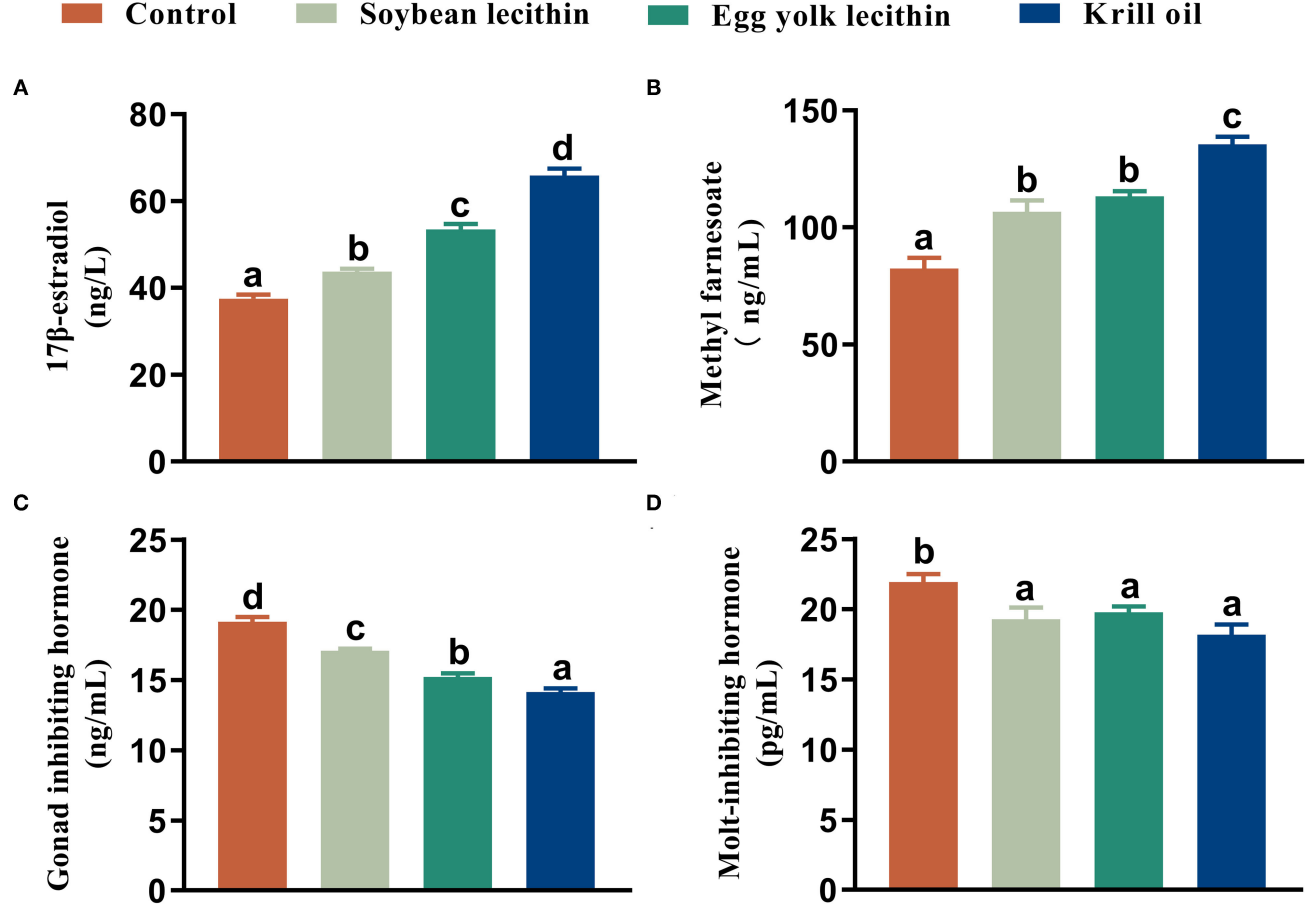
Content of reproductive hormone secretion female *L. vannamei* fed with different experimental diets. **(A)** 17 β-estradiol content in serum. **(B)** Methyl farnesoate content in serum. **(C)** Gonad inhibiting hormone content in eyestalk. **(D)** Molt-inhibiting hormone content in eyestalk. The values are the mean ± standard errors (*n* = 4). Bars with different superscripts are significantly different (*p* < 0.05, single factor analysis of variance and Duncan's tests).

The 17 β-estradiol and methyl farnesoate concentrations in serum were significantly increased in shrimp that was fed with the phospholipid supplement diets compared to the control (*p* < 0.05, Figures 5A,B), and the highest value was found in the shrimp fed with the diet with krill oil. Compared to the control, the GIH and MIH concentrations in the eyestalk of shrimp were significantly reduced when phospholipids were supplemented in the diet (*p* < 0.05, Figures 5C,D). The lowest content of gonad-inhibiting hormone was shown in the shrimp fed with the krill oil diet (*p* < 0.05, Figure 5C). No significant difference was found in the molt-inhibiting hormone contents among the three phospholipid supplement treatments (*p* < 0.05, Figure 5D).

### Lipidomic Analysis of Ovary

**Figure 6:**
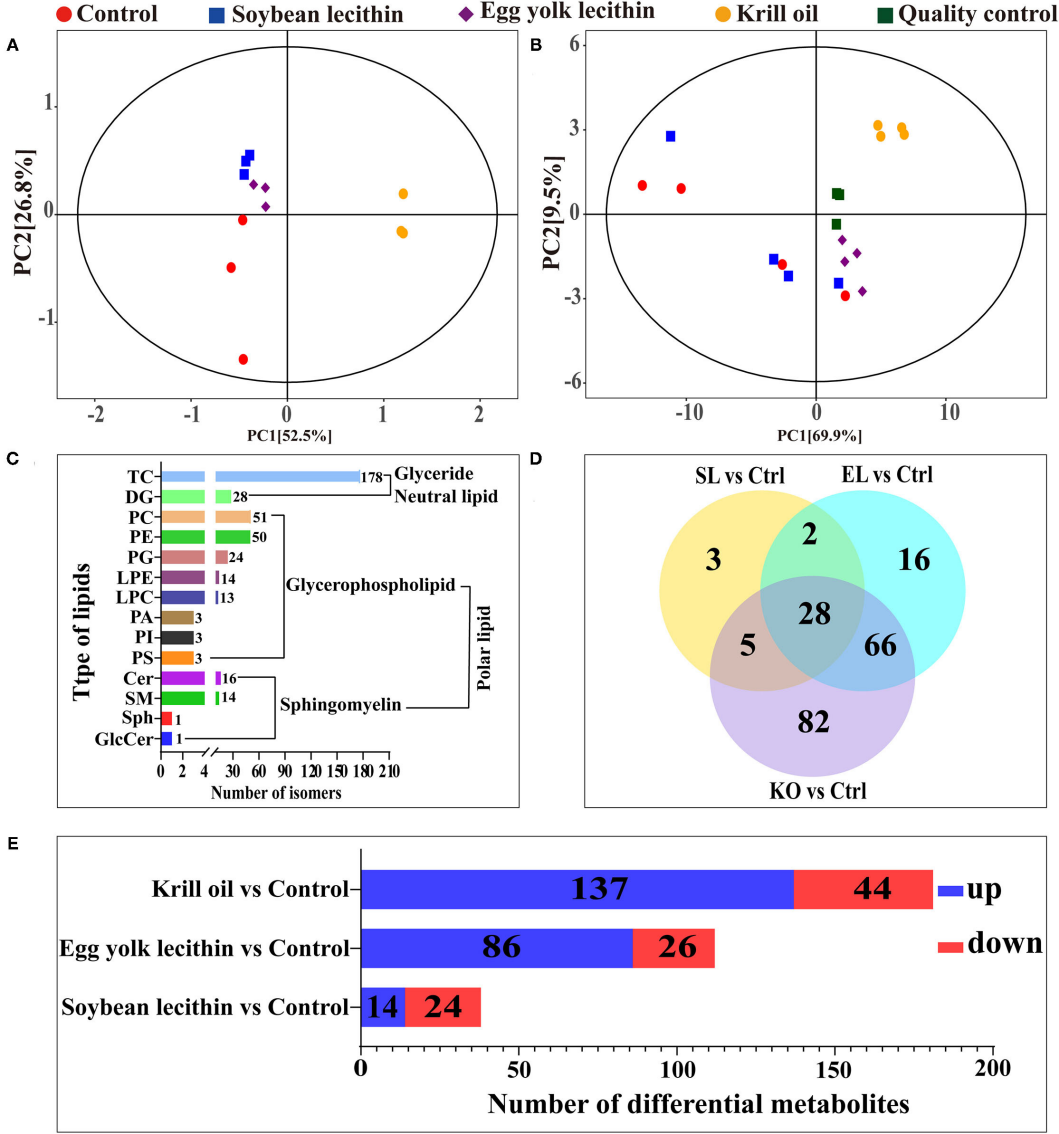
Ovarian lipidomic analysis of different phospholipid source diets. **(A)** Total dispersion point diagram of the principal component analysis (PCA) model for the experimental diet (*n* = 3). **(B)** Score scatter plot for the TOTAL PCA model with quality control of ovarian tissue (*n* = 4). **(C)** Amount of (sub)class within the entire lipidome in ovarian tissue. There are one neutral lipid (glyceride) and two polar lipids (glycerophospholipid and sphingomyelin). **(D)** Venn plot of differential lipid metabolites at the comparison between each treatment group and the control group. **(E)** The number of up- and downregulated differential lipid metabolites was identified at the comparison between each treatment group and the control group. Ctrl (control, phospholipid-devoid), soybean lecithin (SL; added 4% SL), egg yolk lecithin (EL; added 4% EL), krill oil (KO; added 4% KO).

From the results of PCA, it was observed that all the sample confidence intervals were within 95% (Figure 6B) and showed a significant difference among the four experimental treatments in both positive and negative ionization modes. A similar result was also observed in the principal component analysis of the four experimental diets (Figure 6A). A total of 510 peaks (lipids) were extracted from 16 ovarian tissue samples under two ion modes, and 399 peaks were retained after pretreatment. In all lipid species, 178 triacylglycerol (TG), 51 phosphatidylcholine (PC), 50 PE, 28 diacylglycerol (DG), 24 phosphatidylglycerol (PG), 16 ceramide (Cer), 14 lysophosphatidylethanolamine (LPE), 14 sphingomyelin (SM), 13 lysophosphatidylcholine (LPC), 3 phosphatidic acid (PA), 3 PI, 3 phosphatidylserine (PS), 1 sphingosine (Sph), and 1 glucosylceramide (GlcCer) were detected (Figure 6C). Compared with the control, 38, 112, and 181 significantly different metabolites were identified in the soybean lecithin, egg yolk lecithin, and krill oil treatments, respectively (VIP > 1, *p* < 0.05) (Figure 6D). Among these metabolites, 14 upregulated metabolites and 24 downregulated metabolites were identified in a shrimp fed with the soybean lecithin diet, 86 upregulated metabolites and 26 downregulated metabolites were found in a shrimp fed with the egg yolk lecithin diet, and 137 upregulated metabolites and 44 downregulated metabolites were identified in a shrimp fed with the krill oil diet (Figure 6E).

**Figure 7:**
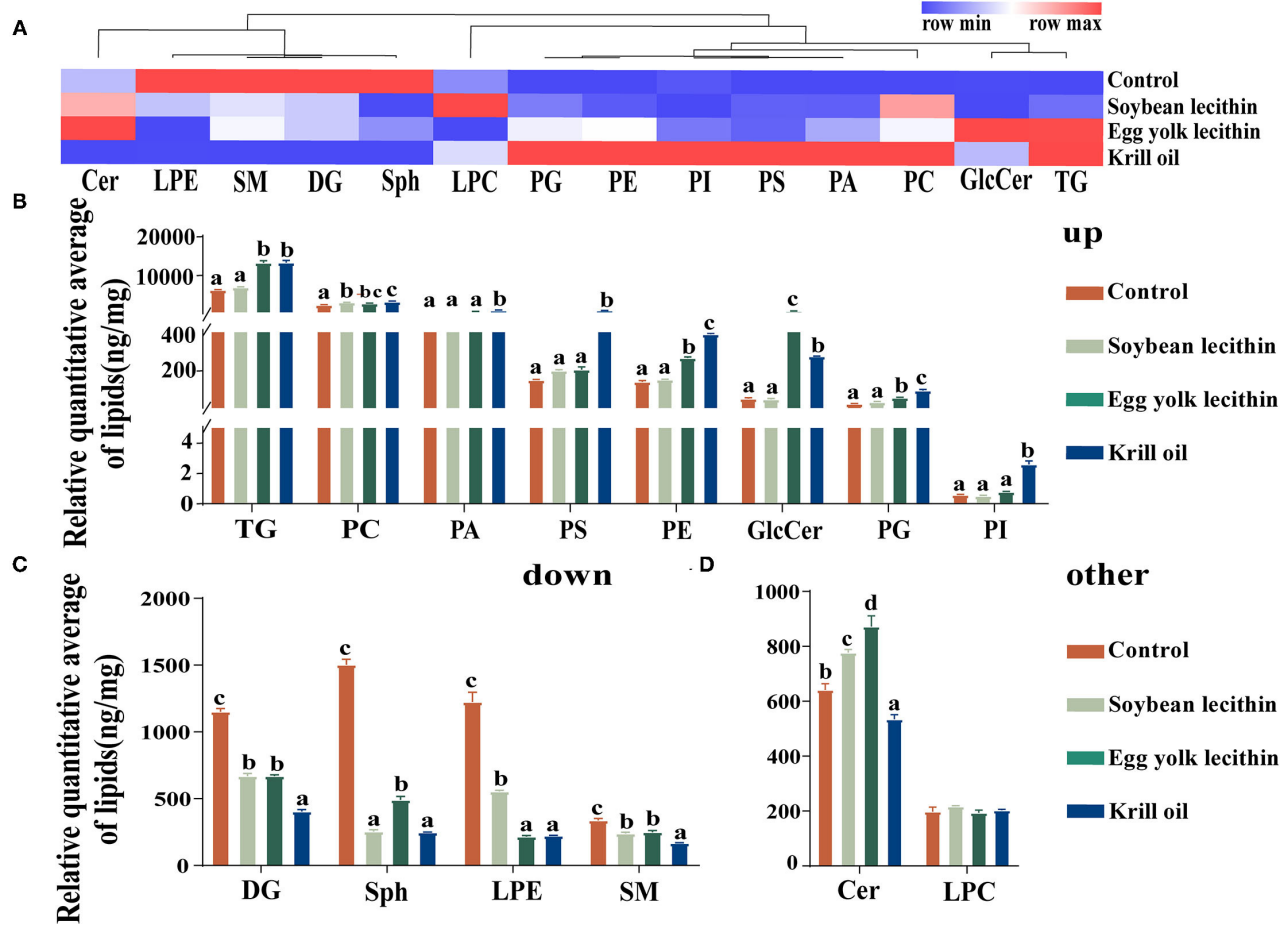
Analysis of lipid differences in ovary. **(A)** Hierarchical cluster analysis of the sum of sublipids in each group. **(B)** Differential analysis of the sum of upregulated sublipids in each group. **(C)** Differential analysis of the sum of downregulated sublipids in each group. **(D)** Differential analysis of the sum of the other sublipids in each group. The values are the mean ± standard errors (*n* = 4). Bars with different superscripts are significantly different (*p* < 0.05, single factor analysis of variance and Duncan's tests).

Different classes of lipids in the ovary are presented in the heatmap for visualization (Figure 7A), and a histogram with a statistical analysis of these differential metabolites among groups was conducted (Figures 7B,D). Quantitative heatmaps of different lipid molecules are presented to reflect the changes among different phospholipid diet treatments (Supplementary Figure S1). Phospholipid supplementation in the diet upregulated the contents of TG, PC, PA, PS, PE, GlcCer, PG, and PI in ovarian tissue (*p* < 0.05, Figure 7B) but downregulated the deposition of DG, Sph, LPE, and SM in the ovary of *L. vannamei* (*p* < 0.05, Figure 7C). In addition, compared with the control, ceramide in the ovary of shrimp fed with diets of soybean lecithin and egg yolk lecithin were significantly increased, while the content was significantly decreased than control in a shrimp fed with a diet of krill oil (*p* < 0.05, Figure 7D). It is worth noting that the contents of PA, PS, PE, PG, and PI were all highest in a shrimp fed with a diet of krill oil.

### Transcriptome Analysis of Ovary

**Figure 8:**
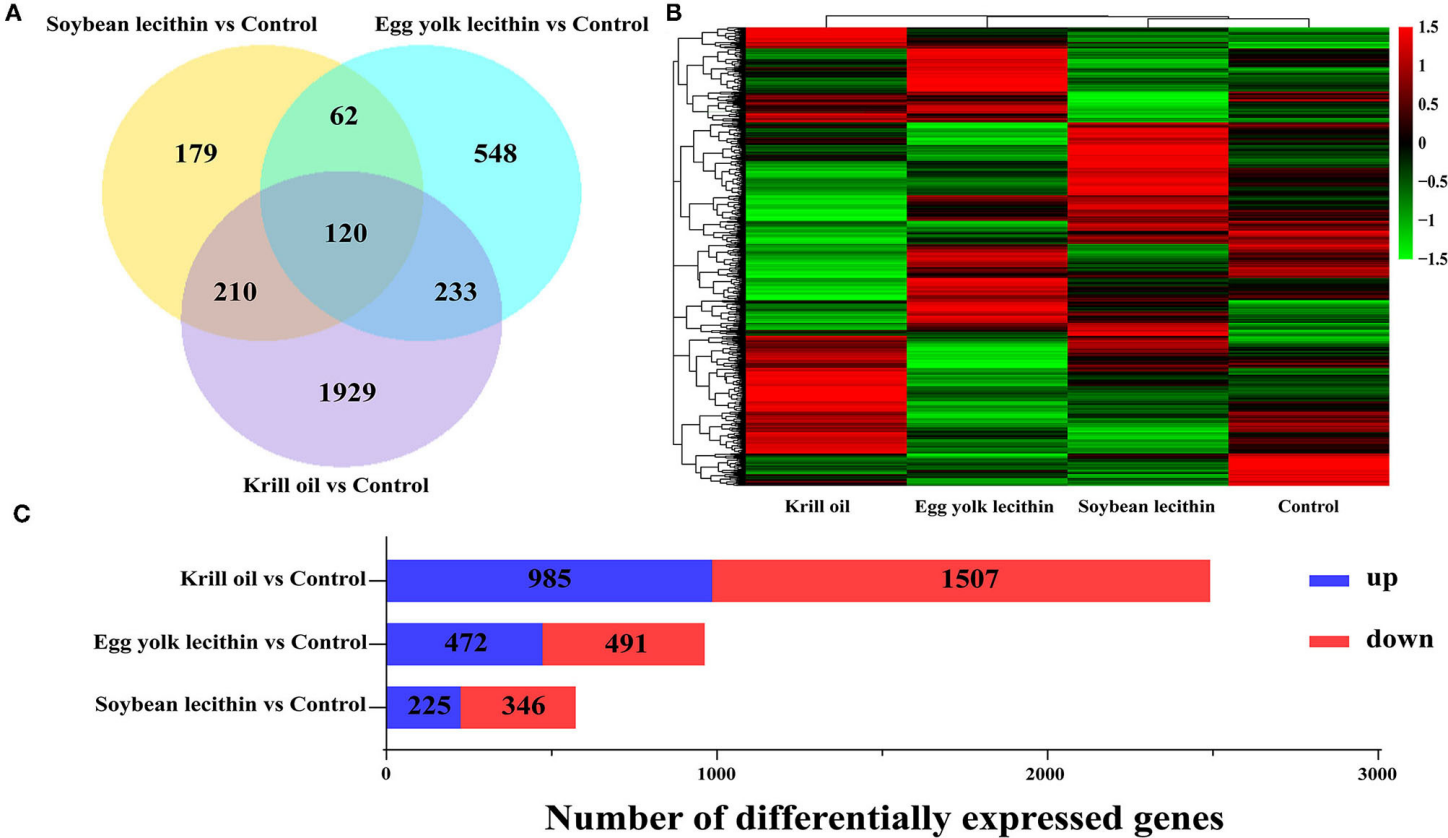
Statistics of differentially expressed genes fed with different phospholipid ovaries. **(A)** Venn plot of differentially expressed genes at the comparison between each treatment group and the control group. **(B)** Gene expression pattern clustering heatmap. Each column in the figure represents a sample, and each row represents a gene. The color in the figure represents the expression value of the gene after standardized treatment in each sample. Red represents a higher expression level of the gene in the sample, and green represents a lower expression. **(C)** The number of up and downregulated differentially expressed genes were identified at the comparison between each treatment and the control.

A total of 3,281 differentially expressed genes (fold change >1, *p* < 0.05) were identified in the ovaries of shrimps fed with a diet of soybean lecithin, egg yolk lecithin, and krill oil compared to the control (Figure 8A). Compared to the control, in the soybean lecithin diet treatment, a total of 571 differentially expressed genes were identified, among which, 225 genes were upregulated and 346 genes were downregulated. In the egg yolk lecithin group, a total of 963 differentially expressed genes were identified, among which, 472 genes were upregulated and 491 genes were downregulated. In the krill oil group, a total of 2,492 differentially expressed genes were identified, among which, 985 genes were upregulated and 1,507 genes were downregulated (Figure 8C). A cluster analysis was performed on all the differentially expressed genes, and a functional enrichment analysis was performed on these genes in each cluster (Figure 8B).

**Figure 9:**
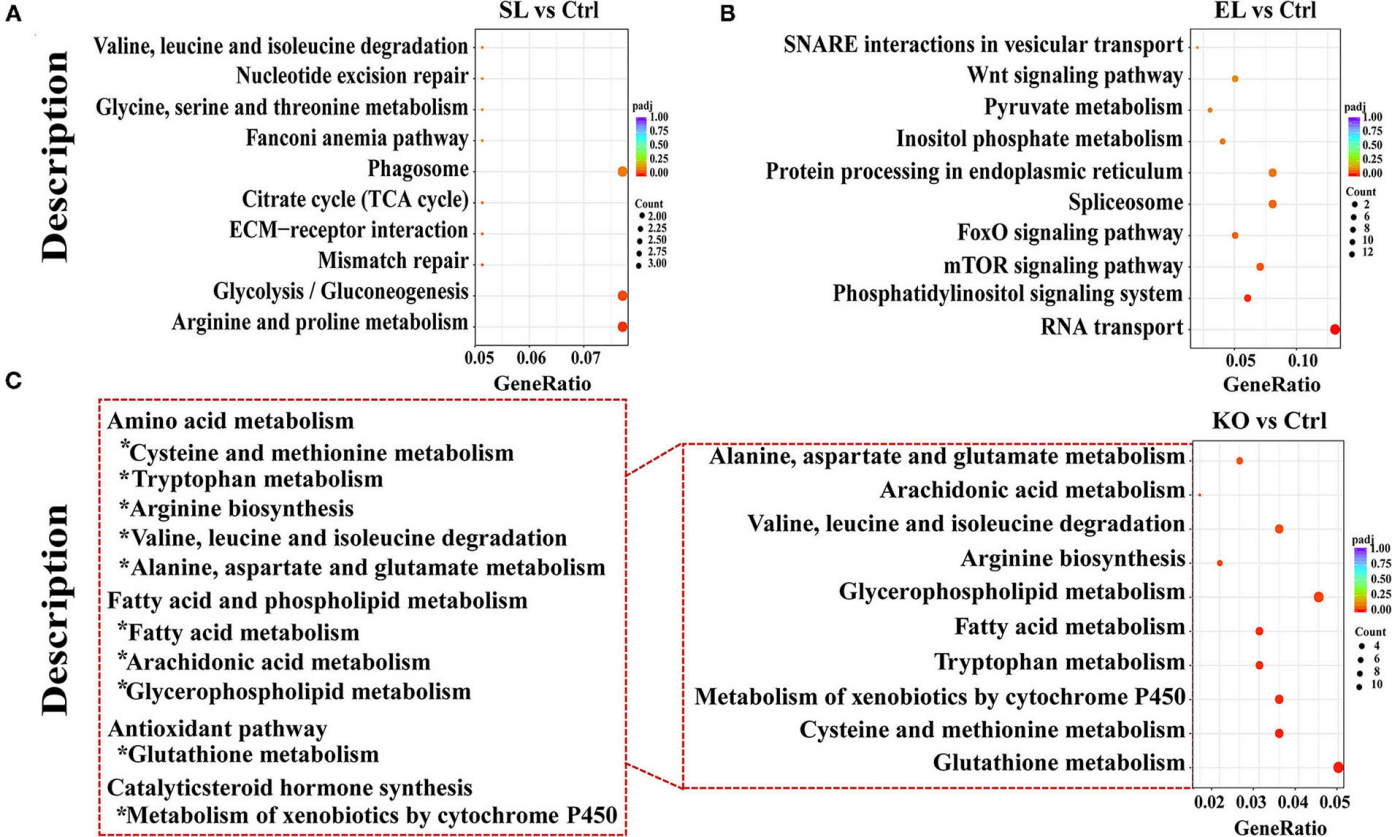
The KEGG pathway analysis of differentially expressed genes. Pathway enrichment analysis plots (top 10) of upregulated differentially expressed genes. **(A)** Top 10 enrichment pathways in SL. **(B)** Top 10 enrichment pathways in EL. **(C)** Top 10 enrichment pathways in krill oil. P-adjusted <0.05 was regarded as a significant enrichment in the relevant term. The gene ratio represents the ratio of the number of differentially expressed genes related to a pathway to the total number of differentially expressed genes. Ctrl (control, phospholipid-devoid), SL (added 4% soybean lecithin), EL (added 4% egg yolk lecithin), and KO (added 4% krill oil).

**Figure 10:**
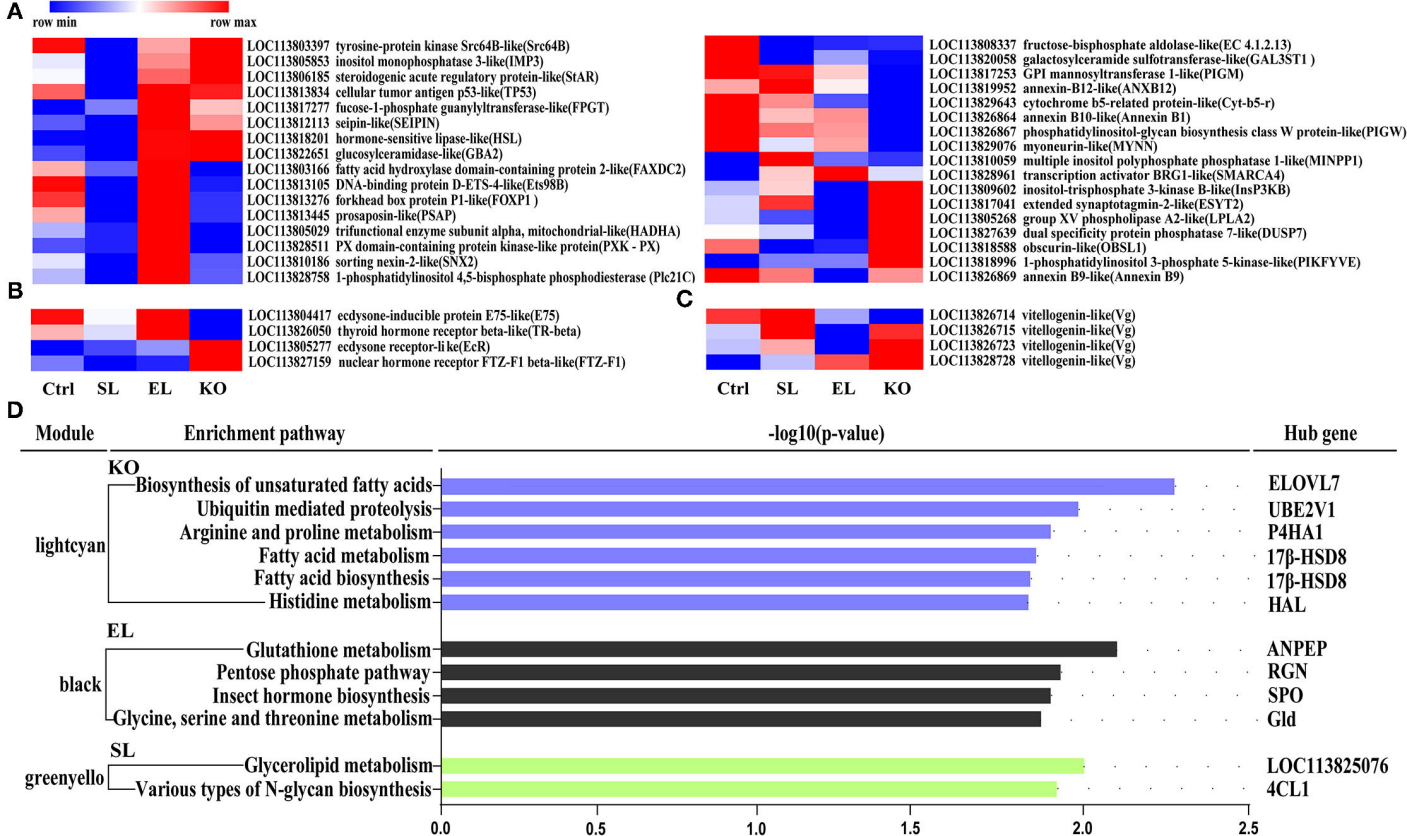
Gene expression (FPKM) clustering heatmap of lipid-related genes **(A)**, hormone-related genes **(B)** and reproduction-related genes **(C)**. Each column in the figure represents a group, and each row represents a gene. Colors from dark blue to dark red indicate the contents of lipid molecules from min to max within a row. **(D)** Pathway enrichment analysis plots of the hub genes with the strongest connectivity in the module. Enrichment differences are expressed in terms of –log10 (p-value). The Lightcyan module is derived from the krill oil group, the black module is derived from the EL group, and the green-yellow module is derived from the SL group. The pathway on the left is the core gene enrichment pathway on the right. Ctrl (control, phospholipid-devoid), SL (added 4% soybean lecithin), EL (added 4% egg yolk lecithin), and KO (added 4% krill oil).

Results of pathway analysis showed that in the top 10 enrichment pathways of upregulated genes in the soybean lecithin group, “arginine and proline metabolism” and “glycolysis/gluconeogenesis” pathways were significantly enriched (*p* < 0.05, Figure 9A). Among the top 10 enrichment pathways of upregulated genes in the egg yolk lecithin group, the “phosphatidylinositol signaling system” and “mTOR signaling pathway” pathways were significantly enriched (*p* < 0.05, Figure 9B). Among the top 10 enrichment pathways of upregulated genes in the krill oil group, “glutathione metabolism” (ID: pvm00480) and amino acid pathways, including “cysteine and methionine metabolism,” “tryptophan metabolism,” “arginine biosynthesis,” “valine, leucine, and isoleucine degradation,” and “alanine, aspartate, and glutamate metabolism,” were significantly enriched. In addition, “fatty acid metabolism,” “glycerophospholipid metabolism,” and “arachidonic acid metabolism” were identified (*p* < 0.05, Figure 9C). The results of weighted gene coexpression network analysis (WGCNA) are shown in Figure 10D. In the green–yellow module of the soybean lecithin group, the hub gene “*LOC113825076*” was involved in “glycerolipid metabolism,” and “4-coumarate-CoA ligase 1-like (*4CL1*)” was involved in “various types of N-glycan biosynthesis.” In the black module of the egg yolk lecithin group, the hub gene “aminopeptidase N-like (*ANPEP*)” was involved in “Glutathione metabolism,” and the “*LOC114251504*” was involved in “Insect hormone biosynthesis.” In the light cyan module of the krill oil group, the hub gene “elongation of very long-chain fatty acids protein 7-like (*ELOVL7*)” was involved in the “biosynthesis of unsaturated fatty acids,” and the “estradiol 17-beta-dehydrogenase 8-like (*17*β*-HSD8*)” was involved in “fatty acid biosynthesis” and “fatty acid metabolism.”

Based on the functional gene annotation, we further screened the lipid, hormone, and reproduction-related genes from differentially expressed genes (Figures 10A–C). Thirty-three lipid metabolism-related genes included “fatty acid hydroxylase domain-containing protein 2-like (*FAXDC2*)” and “tyrosine-protein kinase Src64B-like (*Src64B*).” Hormone-related genes and reproduction-related genes were found, and these included “ecdysone-inducible protein E75-like (*E75*)”, “ecdysone receptor-like (*EcR*)”, and “vitellogenin-like (*Vg*)”. Specifically, the mRNA expression levels of hormone-sensitive lipase-like (*HSL*) and group XV phospholipase A2-like (*LPLA2*) were significantly upregulated in a shrimp fed with a krill oil diet compared with the control. Moreover, the ecdysone receptor-like (*EcR*) and Vg-like genes were also significantly upregulated.

### Correlation Analysis of Metabolic and Transcriptomic Profiles

**Figure 11:**
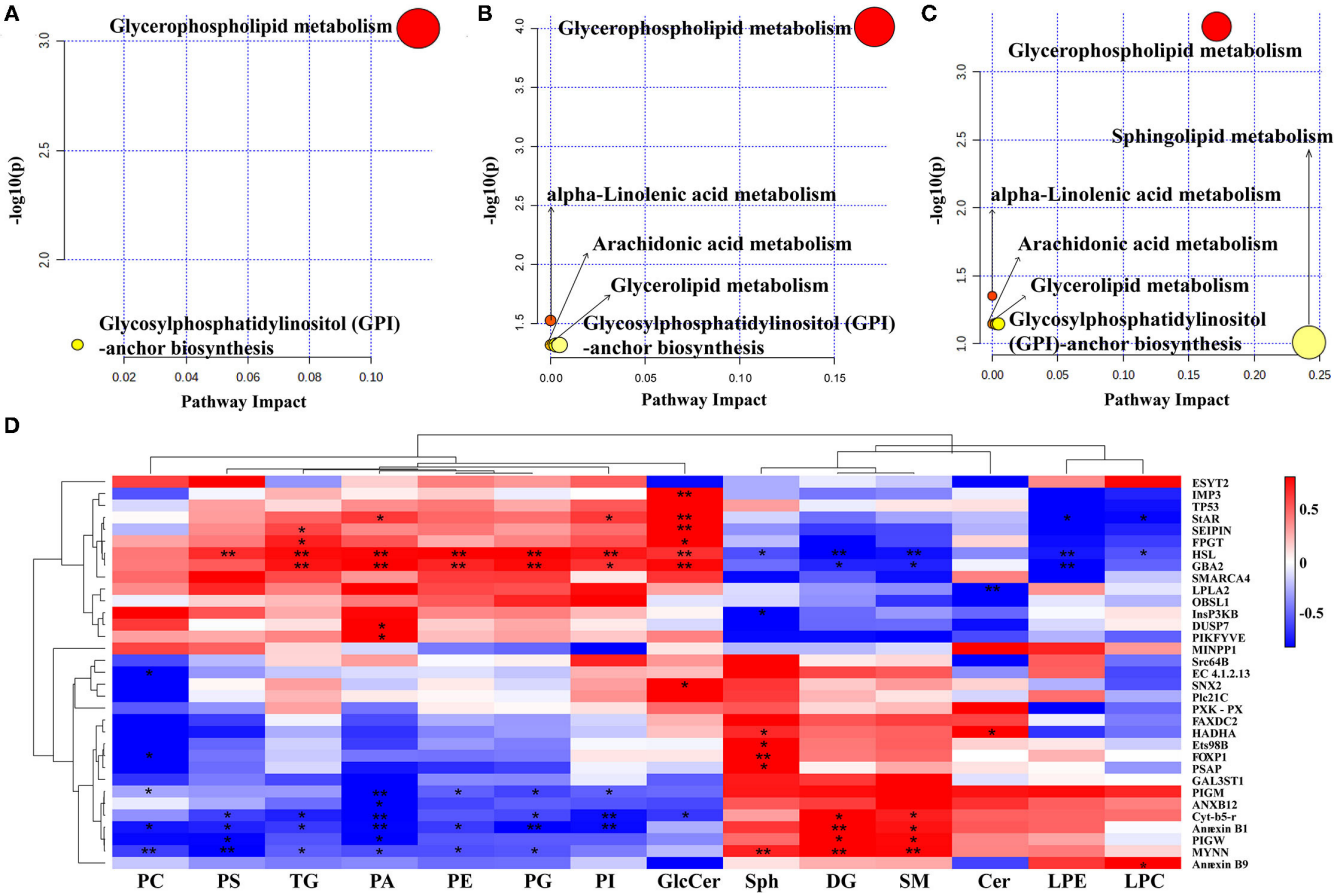
Comprehensive analysis of lipidomic and transcriptomic analysis. Pathway analysis of the differential metabolites. **(A)** Enriched pathway of control vs. SL. **(B)** Enriched pathway of control vs. EL. **(C)** Enriched pathway of control vs. KO. **(D)** Correlation analysis between differential metabolites and differentially expressed genes. Red squares are positive correlations, while blue squares are negative correlations, and the statistical significance is indicated by *(*p* < 0.05), **(*p* < 0.01).

Compared to the differential metabolites of shrimps between the control and three phospholipid groups, the most enriched pathway in common was the “glycerophospholipid metabolism” and “glycosylphosphatidylinositol (GPI)-anchor biosynthesis” (PIV >0.05, Figures 11A–C). The metabolic pathways of “alpha-linolenic acid metabolism,” “arachidonic acid metabolism,” and “glycerolipid metabolism” were all identified in egg yolk lecithin and krill oil experimental treatments (PIV < 0.05, Figures 11B,C). Based on the transcriptome information, the differentially expressed genes in the krill oil group were also enriched in the “arachidonic acid metabolism” pathway (Figure 9C). The “sphingolipid metabolism” pathway was only identified in a shrimp fed with a diet with krill oil (PIV >0.05, Figure 11C).

Spearman's correlation analysis results are shown in Figure 11D. According to the statistics, *HSL* showed a significant positive correlation with 7 differential metabolites, including TG, PA, PS, PE, GlcCer, PG, and PI, but showed a significant negative correlation with 5 differential metabolites, DG, Sph, LPE, SM, and LPC. Glucosylceramidase-like (*GBA2*) showed a significant positive correlation with 6 differential metabolites, including TG, PA, PE, GlcCer, PG, and PI, but a significant negative correlation with three differential metabolites, including DG, LPE, and SM. Annexin B10-like (*AnxB10*) showed a significant positive correlation with 2 differential metabolites, DG and SM, but a significant negative correlation with 7 differential metabolites, TG, PC, PA, PS, PE, PG, and PI. Moreover, *PLA2* showed a significant negative correlation with Cer.

**Figure 12:**
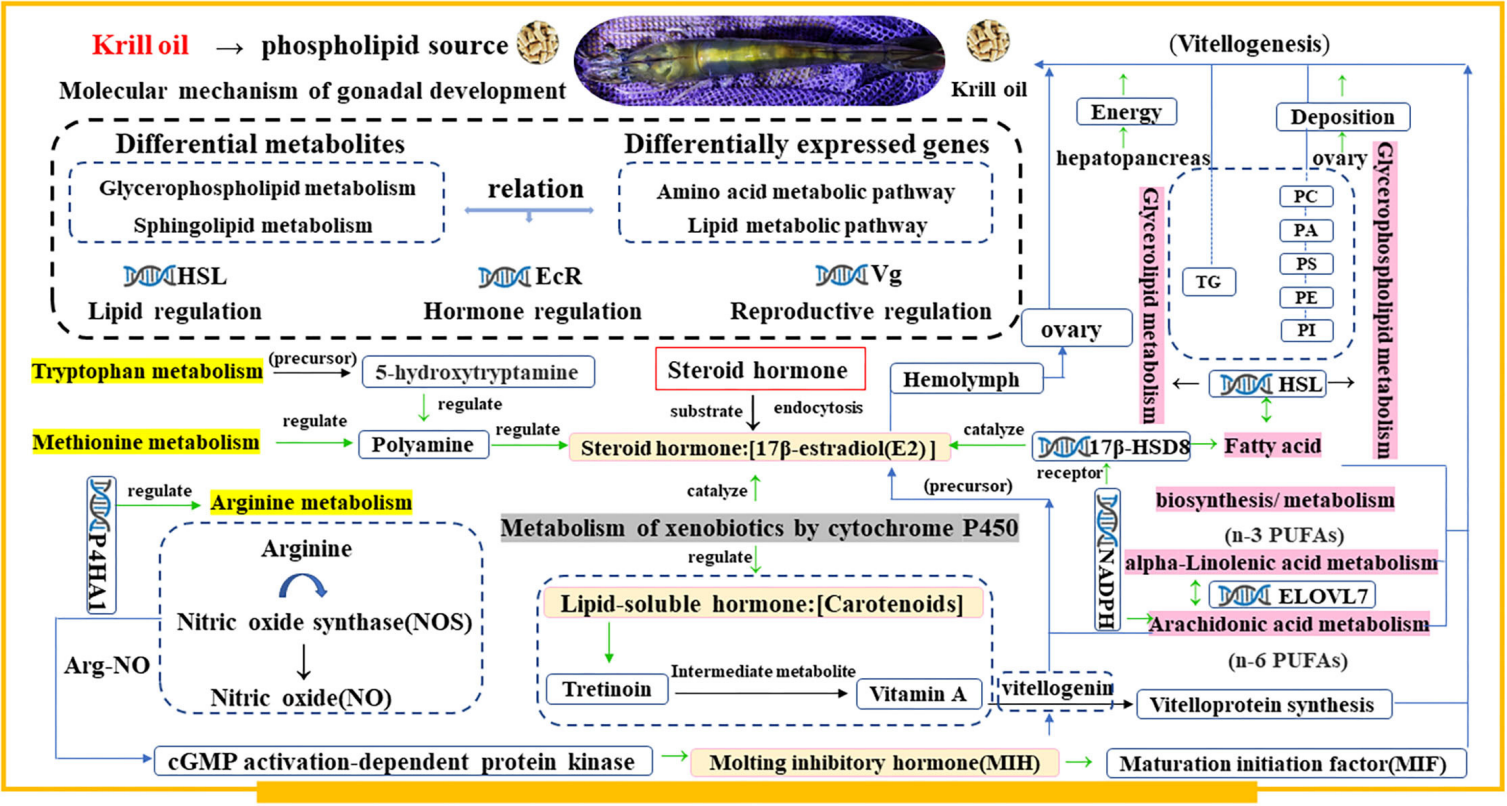
A hypothetical integrated metabolic network of key differential metabolites and genes in significant pathways identified by multiomics analysis after ingestion of krill oil as a phospholipid source of feed based on the KEGG pathway database. The yellow frame represents the metabolism of amino acids. The purple bottom frame represents the metabolism of lipids. The gray bottom box indicates another accessory pathway metabolism. The pink bottom frame represents hormones. The gene symbol represents the hub gene of the pathway. The green arrow indicates the regulation of the downstream. The blue arrow indicates the path direction.

Based on the analysis of all the results, we propose that lipid mobilization, lipid metabolism, and amino acid metabolism jointly participated in the synthesis of sterol hormones and ultimately promoted the vitellogenesis and ovarian development in the shrimp (Figure 12).

## Discussion

Optimal broodstock nutrition is essential for the gonadal maturation of shrimp, and it is considered to be central to a successful reproduction (10). Phospholipids are the main components found in the ovarian tissue of shrimp (14), but the synthesis efficiency of phospholipids is extremely low and cannot meet the nutritional requirements for gonadal maturation in shrimp (16). Therefore, phospholipid supplementation in the diet is necessary to meet the nutritional requirements for gonadal maturation. Based on previous studies, a 4% soybean lecithin content can contribute to the gonadal development and maturation of *L. vannamei* (20). The overall hormone, transcriptome, and lipidomic results in the ovary of *L. vannamei* showed that krill oil is more beneficial for ovarian development maturation than soybean lecithin and egg yolk lecithin diets. Similar results were also found in a study of *E. sinensis*, which showed that krill oil is the best dietary phospholipid choice for gonadal maturation (23).

During the gonadal maturation of crustaceans, lipids are vital fuel for the biosynthesis process of yolk substances and can be absorbed by the developing oocytes (30). The hepatopancreas plays an important role in lipid metabolism and storage in crustaceans (31), and the stored lipids are transported through the hemolymph to the gonads for ovarian development (32, 33). The presence of a very low-density lipoprotein in serum is mainly responsible for the triglyceride transport from the hepatopancreas to peripheral tissue (24). The present study showed the highest gonadosomatic index and a shorter latency period in broodstock shrimp fed with the diet with krill oil, which coincided with the highest serum total cholesterol and very low-density lipoprotein concentrations of shrimp in this treatment. This result indicates increased lipid mobilization from the hepatopancreas to the ovary, as previously reported in *Marsupenaeus japonicas* (34) and *E. sinensis* (18) female broodstock. Lipidomics analysis in this study further confirmed that as the main component of neutral lipids, the content of triglycerides (TGs) in the ovaries of shrimps fed with diets of egg yolk lecithin and of krill oil has increased significantly, and the highest content was found in the krill oil treatment. In contrast, in the research of *L. vannamei* (20) and *Scylla paramamosain* (35), soybean lecithin can significantly increase the content of TG in the hepatopancreas. In this study, the *HSL* gene, which has the function of hydrolyzing various esters (36) and mobilizing the storage of lipids (37), showed a significant positive correlation with ovarian TG content, and the mRNA expression of *HSL* in the ovary of a shrimp fed with a diet of krill oil was the highest among all treatments. Based upon these results, egg yolk lecithin and krill oil in the diet could promote the transport of TG from the hepatopancreas to the ovary more efficiently than soybean lecithin by participating in the formation of lipoprotein.

Meanwhile, the phosphatidylcholine (PC) content in the ovary tissue of a shrimp fed with the krill oil diet was the highest in this study. Previous studies have hypothesized that the PC in dietary phospholipids may act as an acyl donor for lecithin cholesterol acyltransferase, which converts the free cholesterol into a sterol ester (38). Similar results were also observed in female *P. trituberculatus*, which could indirectly confirm the role of phospholipids in cholesterol and triglyceride transport (33). In addition, lipid mobilization can provide energy for organisms and further convert them to steroids and facilitate the Vg (13, 24). Although soybean lecithin and egg yolk lecithin contain a high content of PC (39, 40), krill oil has a richer phospholipid composition and a higher bioavailability (41, 42). Therefore, the krill oil supplementation in the diet was more beneficial to promote the lipid utilization and to provide energy for ovarian development than the soybean lecithin and egg yolk lecithin for shrimp.

The ovary development of shrimp is controlled by a complex and sophisticated neuroendocrine regulatory network, and the oocyte development and vitellogenesis are both regulated by reproductive hormones (43). Steroid hormones have been proven to play a key role in reproductive endocrine regulation (44, 45). Among these sex steroid hormones, estrogen shows an important physiological effect on gonadal development and reproductive behavior, especially in female individuals (46). Among these estrogens, the most active female hormone is estradiol (47). In this study, a shrimp fed with a diet with krill oil showed an increased estrogen secretion but less gonadal inhibitory hormone synthesis. The 17β-Estradiol (E_2_) is the main estrogen that can induce vitellin precursor (Vg) synthesis during liver and oocyte development (48). As the precursor of vitellin, Vg can bind estradiol at its lipid binding site and then be transported from the hepatopancreas to the ovary through hemolymph (49). Based on this, krill oil can promote vitellogenesis better than soy lecithin and egg yolk lecithin for female *L. vannamei*. The results of ovary histology further demonstrated that krill oil could promote a yolk granule deposition in the ovary more efficiently than the other phospholipid sources and controls.

In crustaceans, the synthesis of Vg is regulated by various endocrine factors (50). One of the endocrine factors is methyl farnesoate (MF), a sesquiterpenoid hormone that is secreted by mandibular organs (51). Previous research has shown that MF can stimulate ovarian maturation and vitellogenesis in crustaceans (52). In this study, a dietary krill oil was more effective than soybean lecithin and egg yolk lecithin in promoting the secretion of MF in *L. vannamei*, thereby promoting ovarian maturation and vitellogenesis. Studies of *L. vannamei* (53) and *Oziotelphusa senex senex* (54) also confirmed this enhancement effect of MF. Meanwhile, the secretion of GIH and MIH from the eyestalk was lowest in a shrimp fed with a diet of krill oil compared with other experimental treatments. Combined with the analysis of the transcriptome and lipidomics, we speculate that the synergistic effect of differential genes (*17*β*-HSDs, CYP450*, and *PLA2*) and differential metabolites (glycerides, glycerophospholipids, a-linolenic acid, and arachidonic acid) in metabolic pathways will ultimately promote the secretion of steroid hormones in *L. vannamei*.

Previous studies have suggested that the possible synthesis sites of estradiol in crustaceans are the ovary and the hepatopancreas, and its synthesis is converted from cholesterol under the action of a series of enzymes (49), including 17β-hydroxysteroid dehydrogenase (*17*β*-HSDs*), aromatase, and 17α-hydroxylase found in crustaceans such as *Marsupenaeus japonicus* (55). In the ovary transcriptome results, the *17*β*-HSDs* of the hub genes were identified in the fatty acid metabolism pathway in a shrimp fed with a krill oil diet. The *17*β*-HSDs* are key factors that catalyze the steroid biosynthesis and metabolism in vertebrates (56), and the regulatory effect on the development of the gonads of marine organisms has also been confirmed (57). The *17*β*-HSDs* can promote the mutual conversion between the active (17-hydroxy) form and the inactive (17-keto) form of specific steroids in an NADPH-dependent manner and participate in sex hormone metabolism (58). In addition, differentially expressed genes in the ovary of a shrimp fed with a krill oil diet were enriched in “metabolism of xenobiotics by cytochrome P450 (*CYP450*).” Aromatase is *CYP450(19)*, the rate-limiting enzyme in estrogen biosynthesis, through interaction with NADPH-cytochrome P450 reductase (CPR) and catalyzes a three-step hydroxylation to convert androgens (androstenedione and testosterone) to estrogen (estrone and estradiol) (59). Therefore, dietary krill oil can promote a steroid hormone secretion, especially E_2_, better than soybean lecithin and egg yolk lecithin, thereby promoting ovarian development.

As mentioned above, CYP450 enzymes can convert androgens to estrogen. Polyunsaturated fatty acids (PUFAs), including arachidonic acid (ARA) and its metabolites, have been shown to regulate the transfer of cholesterol from the outer to the inner membrane of mitochondria. The CYP450 enzyme uses cholesterol as a precursor to initiate steroid hormone synthesis (60). The lipidomics analysis showed the difference in lipid metabolites of a shrimp fed with diets of egg yolk lecithin and of krill oil. The metabolic pathways of “alpha-linolenic acid metabolism,” “arachidonic acid metabolism,” and “glycerolipid metabolism” were identified as significant responses in the ovarian tissue of shrimp by dietary treatments. Based on the ovarian transcriptome information, it was found that the differentially expressed genes in a shrimp fed with a krill oil diet were also enriched in the “arachidonic acid metabolism” pathway. The ARA is the precursor substance of prostaglandins (61) and is considered key in reproducing many aquatic species (62). Phospholipase A2 (*PLA2*) can mobilize ARA from cellular phospholipids (63). Dietary krill oil significantly upregulated *PLA2* mRNA levels in the ovary of shrimp compared with soybean lecithin and egg yolk lecithin in this study. The results showed that krill oil is more able to promote the expression of *PLA2* mRNA and mobilize cellular phospholipids. Then, the free ARA can be oxidized through the CYP450 pathway to generate prostaglandins (64). Prostaglandins can promote ovarian maturation, including vitellogenesis and oviposition, in many decapod crustaceans (65). The prostaglandin concentration in the ovary is highly correlated with yolk development within the ovarian cycle in *M. rosenbergii* (65). Based on the above analysis, ARA metabolism in shrimp was activated after ingesting the krill oil. In addition, related factors participated in prostaglandin synthesis, which may be one of the reasons for ultimately promoting the ovarian development in *L. vannamei*.

In this study, the specific differentially expressed genes in a shrimp fed with a diet of krill oil were also involved with amino acids (tryptophan, methionine, and arginine) related to metabolism. Amino acids play a significant role in various physiological processes. The release of some hormones can be influenced by dietary amino acid intake (66). As one of the essential nutrients in ovarian development, amino acids promote the ovarian maturation and reproduction by promoting the synthesis of yolk proteins, hormone polypeptides, and enzymes. Studies have shown that the addition of arginine to the diet promotes an increase in ecdysterone concentration in the serum of *E. sinensis* (67). In this study, the “arginine metabolism” pathway enriched with significantly differentially expressed genes may participate in regulating MIH secretion through the Arginine-Nitric oxide (Arg-NO) pathway. The nitric oxide synthase (NOS) can catalyze NO synthesis from L-arginine, oxygen, NADPH, and NO and can freely diffuse across the cell membrane to induce reactions in adjacent cells (68). The NOS has been identified in the land crab *Gecarcinus lateralis* with a high homology to the NOS gene sequence in insects and has showed significantly high NOS mRNA expression levels in ovaries, testes, and ophthalmic stalk ganglion (69).

In adults, NOS regulates the ecdysteroid synthesis in the Y-organ in addition to its neuromodulatory function (70). Furthermore, preliminary studies have shown that NO can inhibit the secretion of MIH by regulating the second messenger cGMP-dependent protein kinase activity in shrimp and crabs (70), further promoting the Y-organ to produce ecdysteroids (71) and the synthesis of Vg (72). Because dietary krill oil significantly reduces the MIH secretion of *L. vannamei*, we speculate that the intake of krill oil can promote the metabolism of gonadal development substances and can increase the synthesis of yolk through the Arg-NO pathway. Among tryptophan metabolism pathways, 5-hydroxytryptamine (5-HT) is a monoamine produced by the essential amino acid tryptophan (73) and participates in the regulation of polyamines. In addition, there is evidence that polyamines are involved in follicular development and ovulation in female mammals, and that polyamine synthesis is necessary for steroid production in the ovary (74).

## Conclusion

This study established the relationship between phospholipid nutrition and gonadal development of broodstock female *L. vannamei*. In conclusion, dietary supplementation with phospholipids, especially using krill oil as a phospholipid source, can promote the development of ovarian tissue of *L. vannamei*. The advantages of dietary phospholipid supplementation are mainly reflected in three ways: (1) promoting sterol hormone secretion and enhancing the regulation of hormones on ovarian development; (2) lipid metabolism (glycerides, glycerophospholipids, a-linolenic acid, and arachidonic acid) which enriches ovarian lipid composition, and lipid mobilization which provides energy for ovarian development and promotes yolk accumulation, and (3) amino acid metabolism (tryptophan, methionine, arginine) which also participates in the secretion of steroid hormones to promote ovarian development.

## Data Availability Statement

The datasets presented in this study can be found in online repositories. The names of the repository/repositories and accession number(s) can be found at: NCBI; PRJNA786473.

## Ethics Statement

The animal study was reviewed and approved by the Committee on the Ethics of Animal Experiments of East China Normal University (No. f20201001).

## Author Contributions

XLi contributed to writing original manuscripts, feeding management, and data analysis. XLu and HL contributed to the feeding management. XLi, FH, CX, and EL contributed to the research investigation and experimental design. CX, EL, JQ, and LC contributed to the writing review and revision. EL contributed to the overall planning of funds. All authors contributed to the article and approved the submitted version.

## Funding

This study was sponsored by the Research and Development Program Projects in Key Areas of Guangdong Province [2020B0202010001] and the National Key R&D Program of China [2018YFD0900400].

## Conflict of Interest

The authors declare that the research was conducted in the absence of any commercial or financial relationships that could be construed as a potential conflict of interest.

## Publisher's Note

All claims expressed in this article are solely those of the authors and do not necessarily represent those of their affiliated organizations, or those of the publisher, the editors and the reviewers. Any product that may be evaluated in this article, or claim that may be made by its manufacturer, is not guaranteed or endorsed by the publisher.
