# Supplementary material for: Effects and Mechanism of Different Phospholipid Diets on Ovary Development in Female Broodstock Pacific White Shrimp, Litopenaeus vannamei
Source: Front Nutr. 2022 Feb 18;9:830934. doi: 10.3389/fnut.2022.830934 (PMC8894211; doi:10.3389/fnut.2022.830934)

**Supplementary Table S1**

Fatty acid composition of four experimental diets (μg/g).

| **Fatty acids** | | **Experiment diets** | | | |
| --- | --- | --- | --- | --- | --- |
|  |  | **Control** | **Soybean lecithin** | **Egg yolk lecithin** | **Krill oil** |
| Fatty acids with higher relative content in soybean lecithin group | | | | | |
| cis, cis-9,12-Linoleic Acid | C18:2 | 287.09^a^ | 1524.91^c^ | 493.94^b^ | 317.25^a^ |
| trans-11-Octadecenoic acid | C18:1 | 830.25^b^ | 1205.87^d^ | 1134.90^c^ | 770.11^a^ |
| cis, cis, cis-9,12,15-Linolenic Acid | C18:3 | 31.89^a^ | 173.72^d^ | 41.34^b^ | 69.53^c^ |
| Eicosanoic Acid | C20:0 | 15.30^a^ | 18.36^c^ | 16.53^b^ | 14.79^a^ |
| Lignoceric Acid | C24:0 | 11.08^a^ | 12.64^c^ | 11.74^b^ | 11.41^ab^ |
| Behenic Acid | C22:0 | 7.74^a^ | 10.54^c^ | 8.41^b^ | 8.74^b^ |
| Tricosanoic Acid | C23:0 | 6.03^a^ | 6.92^b^ | 6.23^a^ | 6.11^a^ |
| Fatty acids with higher relative content in egg yolk lecithin group | | | | | |
| Stearic Acid | C18:0 | 344.36^a^ | 452.66^b^ | 545.20^c^ | 337.91^a^ |
| Fatty acids with higher relative content in krill oil group | | | | | |
| all-cis-4,7,10,13,16,19-Docosahexaenoic Acid (DHA) | C22:6 | 271.94^a^ | 298.88^b^ | 307.39^b^ | 1165.66^c^ |
| all-cis-5,8,11,14,17-Eicosapentaenoic Acid | C20:5 | 144.85^a^ | 170.41^b^ | 167.81^b^ | 1030.38^c^ |
| cis-11-Octadecenoic acid | C18:1 | 67.36^a^ | 115.54^c^ | 95.06^b^ | 249.20^d^ |
| Myristic Acid | C14:0 | 109.06^a^ | 148.97^b^ | 147.85^b^ | 229.54^c^ |
| cis-15-Nervonic Acid | C24:1 | 45.92^a^ | 50.32^b^ | 50.74^b^ | 168.17^c^ |
| cis-9-Palmitoleic Acid | C16:1 | 95.84^a^ | 141.61^b^ | 147.52^c^ | 165.60^d^ |
| all-cis-7,10,13,16,19-Docosapentaenoic acid (EPA) | C22:5 | 46.62^a^ | 50.99^b^ | 52.39^b^ | 69.70^c^ |
| cis-11-Eicosenoic Acid | C20:1 | 30.32^a^ | 39.36^c^ | 37.91^b^ | 40.42^d^ |
| Pentadecanoic Acid | C15:0 | 19.47^a^ | 25.29^b^ | 23.77^b^ | 29.57^c^ |
| cis-13-Erucic Acid | C22:1 | 8.47^a^ | 9.57^a^ | 9.39^a^ | 17.48^b^ |
| cis, cis, cis-6,9,12-Linolenic Acid | C18:3 | 9.52^a^ | 10.24^b^ | 10.55^b^ | 12.26^c^ |
| cis, cis, cis-8,11,14-Linolenic Acid | C20:3 | 5.25^a^ | 5.55^a^ | 6.68^b^ | 7.98^c^ |
| all-cis-11,14,17-Eicosatrienoic Acid | C20:3 | 4.64^a^ | 5.07^b^ | 5.22^b^ | 7.98^c^ |
| trans-13-Docosenoic acid | C22:1 | 0.00^a^ | 0.00^a^ | 0.00^a^ | 5.87^b^ |
| Fatty acids with no significant difference in pairwise comparison | | | | | |
| Palmitic Acid | C16:0 | 1167.74^a^ | 1901.81^c^ | 1867.66^c^ | 1556.67^b^ |
| all-cis-5,8,11,14-Eicosatetraenoic Acid (AA) | C20:4 | 32.06^a^ | 35.39^b^ | 66.35^c^ | 63.25^c^ |
| Heptadecanoic Acid | C17:0 | 23.35^a^ | 28.91^c^ | 28.25^c^ | 26.42^b^ |
| trans-9-Elaidic Acid | C18:1 | 13.76^ab^ | 13.23^a^ | 14.03^b^ | 14.36^b^ |
| cis, cis-11,14-Eicosadienoic Acid | C20:2 | 7.45^a^ | 9.06^b^ | 10.45^b^ | 10.26^b^ |
| Lauric Acid | C12:0 | 7.41^a^ | 8.72^a^ | 12.11^b^ | 9.90^ab^ |
| trans, trans-9,12-Linolelaidic Acid | C18:2 | 6.94^b^ | 6.85^ab^ | 6.90^ab^ | 6.65^a^ |
| tran-9-Palmitelaidic acid | C16:1 | 4.96^ab^ | 4.90^ab^ | 4.57^a^ | 5.08^b^ |
| Heneicosanoic Acid | C21:0 | 3.68^a^ | 3.99^c^ | 3.77^ab^ | 3.94^bc^ |
| cis-9-Myristoleic Acid | C14:1 | 3.61^a^ | 4.16^b^ | 4.43^b^ | 4.57^b^ |
| trans-11-Eicosenoic acid | C20:1 | 2.44^b^ | 2.54^b^ | 1.95^b^ | 0.36^a^ |
| Saturated fatty acid | SFA | 1715.21^a^ | 2609.29^c^ | 2488.04^bc^ | 2234.98^b^ |
| Monounsaturated fatty acid | MUFA | 1133.36^a^ | 1615.88^d^ | 1529.36^c^ | 1470.01^b^ |
| Polyunsaturated fatty acid | PUFA | 690.57^a^ | 995.44^b^ | 1971.61^d^ | 1679.64^c^ |
| n-3 fatty acid | n-3 FA | 495.31^a^ | 694.01^c^ | 568.93^b^ | 2335.27^d^ |
| n-6 fatty acid | n-6 FA | 335.62^a^ | 1577.41^d^ | 577.74^c^ | 399.4^b^ |
| n-9 fatty acid | n-9 FA | 106.22^a^ | 152.33^b^ | 158.08^c^ | 176.77^d^ |

The values are the mean ± standard errors (n=4). Different superscripts (a, b, c and d) in the same row represent significant difference (*P* <0.05,

single factor analysis of variance and Duncan's tests).

**Supplemental Figure S1︱**Heatmaps for hierarchical clustering analysis of differential metabolites in ovary. Colors from dark blue to dark red indicate the contents of lipid molecules from min to max within a row. Ctrl (control group, phospholipid-devoid), SL (added 4% soybean lecithin), EL (added 4% egg yolk lecithin), KO (added 4% krill oil).


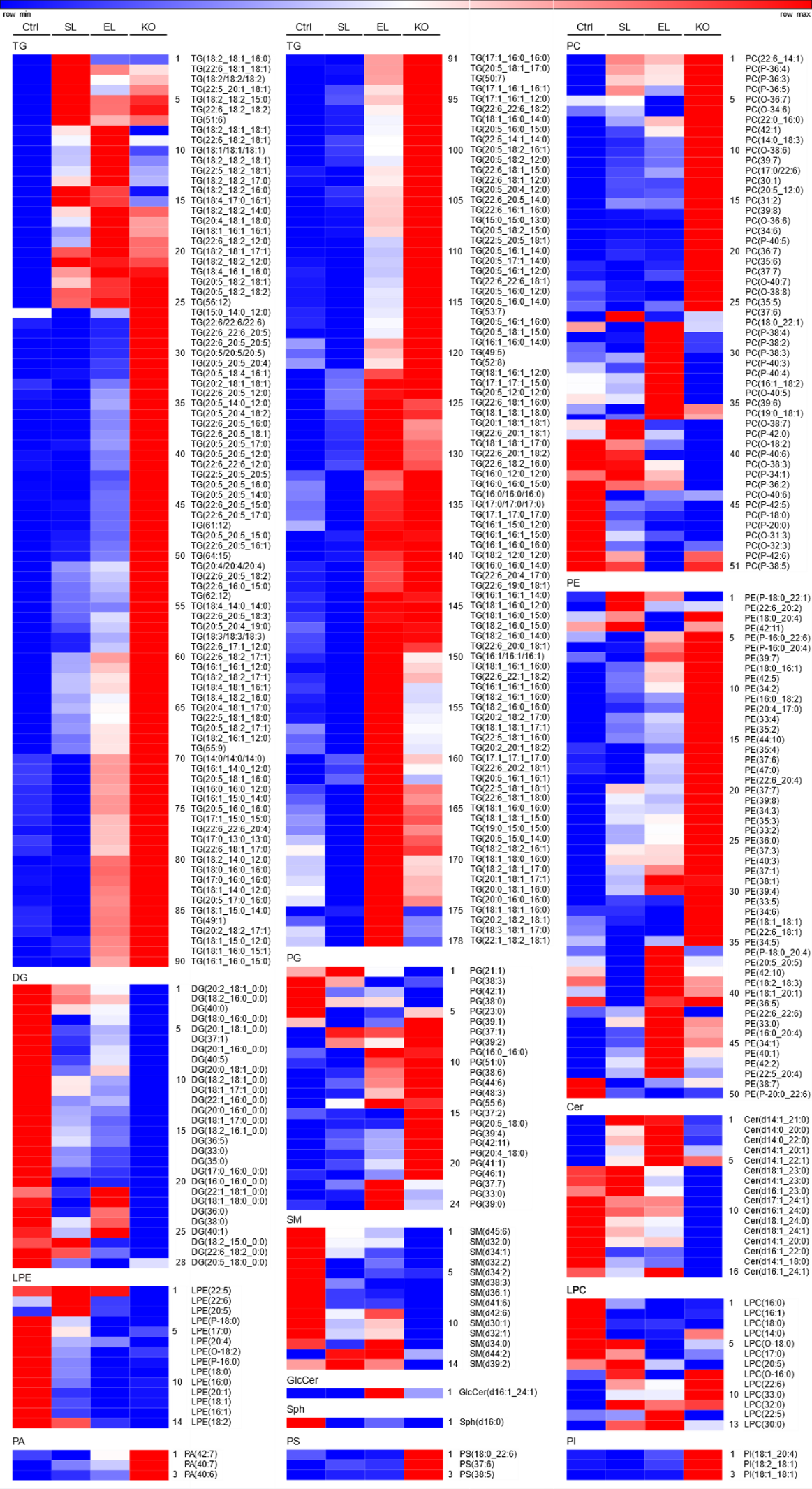


**Supplemental Figure S2︱**Dispersion point diagram of PCA model for ovary.


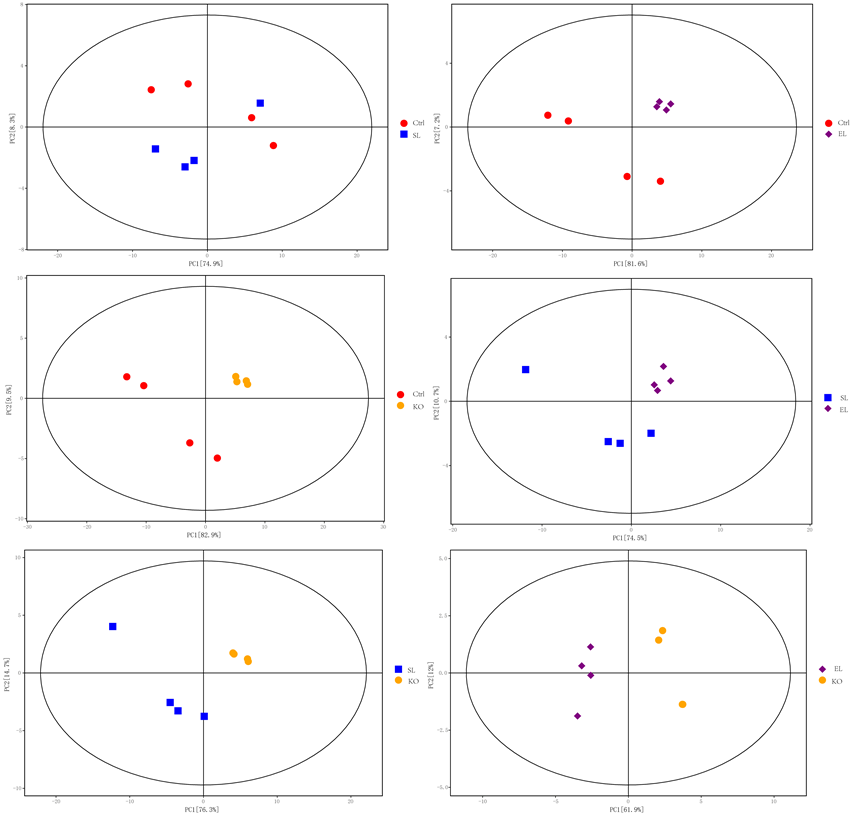

Supplement: Supplementary file 1 [file Data_Sheet_1.docx]
